# 3D bioprinting of high cell-density heterogeneous tissue models through spheroid fusion within self-healing hydrogels

**DOI:** 10.1038/s41467-021-21029-2

**Published:** 2021-02-02

**Authors:** Andrew C. Daly, Matthew D. Davidson, Jason A. Burdick

**Affiliations:** Department of Bioengineering, University of Pennsylvania, Philadelphia, PA USA

**Keywords:** Cardiovascular models, Drug screening, Cardiovascular diseases, Biomedical engineering, Drug delivery

## Abstract

Cellular models are needed to study human development and disease in vitro, and to screen drugs for toxicity and efficacy. Current approaches are limited in the engineering of functional tissue models with requisite cell densities and heterogeneity to appropriately model cell and tissue behaviors. Here, we develop a bioprinting approach to transfer spheroids into self-healing support hydrogels at high resolution, which enables their patterning and fusion into high-cell density microtissues of prescribed spatial organization. As an example application, we bioprint induced pluripotent stem cell-derived cardiac microtissue models with spatially controlled cardiomyocyte and fibroblast cell ratios to replicate the structural and functional features of scarred cardiac tissue that arise following myocardial infarction, including reduced contractility and irregular electrical activity. The bioprinted in vitro model is combined with functional readouts to probe how various pro-regenerative microRNA treatment regimes influence tissue regeneration and recovery of function as a result of cardiomyocyte proliferation. This method is useful for a range of biomedical applications, including the development of precision models to mimic diseases and the screening of drugs, particularly where high cell densities and heterogeneity are important.

## Abstract (web summary)

Cellular models are needed to study disease in vitro and to screen drugs for toxicity and efficacy. Here the authors develop a bioprinting approach to transfer spheroids into self-healing support hydrogels at high resolution, which enables their patterning and fusion into high-cell density microtissues of prescribed spatial organization.

## Introduction

Cells possess the remarkable capacity to self-organize into multicellular spheroids in vitro, including from stem cells and into specialized organoid structures^1^. These spheroids are being used to emulate organs such as the intestine^2^, liver^3^, kidney^4^, brain^5^, and heart^6^ to study human development and disease in vitro. In these formats they are promising as drug screening platforms due to their superior predictability and physiological structure and function when compared to traditional monolayer cultures^7–10^. Indeed, the organotypic cell densities in spheroids enhance the cell-cell and cell-extracellular matrix (ECM) interactions that are required to maintain cellular differentiation and phenotype, whereas these interactions are limited in traditional 2D cultures^11–14^. High cell densities are necessary to accurately recapitulate many pathological disease states such as cancer or fibrosis where perturbed cell-cell and cell-ECM interactions are central to disease progression. For example, spheroid cultures have enabled the engineering of heart, liver, and lung fibrosis models^6,15–18^, and high cell densities within spheroids result in oxygen gradients that mimic the cancer microenvironment^19–21^.

While spheroid cultures hold tremendous potential due to their organotypic cellular densities, it is difficult to control their patterning across larger length scales, to introduce the heterogeneity and resulting function of many tissues. This limits their utility as in vitro models where patterns of cells and ECM are key aspects of development or the progression of disease. For example, during fibrosis of the heart, liver and lung^22,23^, non-functional fibrotic tissue persists within healthy parenchyma, promoting further scarring and eventual organ failure. The development of biofabrication tools to control spheroid assembly across larger length scales through directed placement and fusion could improve their physiological relevance for a range of biomedical applications.

Biofabrication technologies, such as bioprinting, enable programmed assembly of cells into complex 3D geometries^24,25^. However, these technologies have been predominantly designed to process cells embedded within hydrogels, which limits cell-cell interactions and results in low cell density constructs. This has motivated the development of biofabrication technologies tailored specifically for spheroids, often termed bioassembly, which leverages the capacity of spheroids to fuse through liquid-like coalescence to minimize adhesive-free energy at the interface^26–28^. In an early approach, extrusion bioprinting was adopted to allow spheroids to fuse together into larger tissue strands, which could then be extruded through a microcapillary^29^. More recent bioassembly advances enable the direct processing of single spheroids, offering improved control over spheroid patterning and overall geometries. For example, spheroids can be aspirated and then skewered onto supporting metallic needles (i.e., kenzan method) for fusion^30^. Hydrogels have also been used to support aspiration based methods, through the sequential layering of an uncrosslinked hydrogel precursor and spheroids, followed by hydrogel crosslinking^31^. A number of hydrogels have been implemented with this method including an alginate precursor solidified by ionic crosslinking with CaCl_2_ (delivered with a secondary humidifier step), a fibrinogen precursor solidified by enzymatic crosslinking with thrombin (delivered using secondary microvalve based deposition), and a gelatin methacryloyl precursor solidified by radical photocrosslinking with UV light. Despite advances with these bioassembly technologies, significant challenges remain, particularly to support the patterning of complex 3D structures without the need to disrupt spheroids mechanically or to use external stimuli to initiate hydrogel crosslinking. Thus, the development of improved and simplified platforms to pattern spheroids in 3D are needed.

Here, we report a method where spheroids can be translated through a shear-thinning hydrogel that self-heals to receive and hold the spheroid in 3D space, including for the directed fusion between spheroids to form high cell density microtissues with defined shapes that can then be removed from the hydrogel. The self-healing properties of the support hydrogel enable precise positioning of spheroids (up to 10% of spheroid diameter) and high spheroid viability post-printing (~95%), while the viscoelastic and non-adhesive nature of the self-healing hydrogel facilitates controlled fusion between adjacent spheroids into prescribed and stable structures during culture. To demonstrate the utility of this bioprinting method, we develop a cardiac disease model that mimics post-myocardial infarction (MI) scarring, by bioprinting microtissues containing spatially controlled densities of induced pluripotent stem cell (iPSC)-derived cardiomyocytes and primary human cardiac fibroblasts (CF). The bioprinted model replicates cardiac pathologies that arise post-MI (reduced contractile output and electrical synchronization) and we use the model to probe miRNA therapeutics that improve cardiac regeneration and function as a result of cardiomyocyte proliferation.

## Results and discussion

### 3D bioprinting spheroids within self-healing hydrogels

**Fig. 1:**
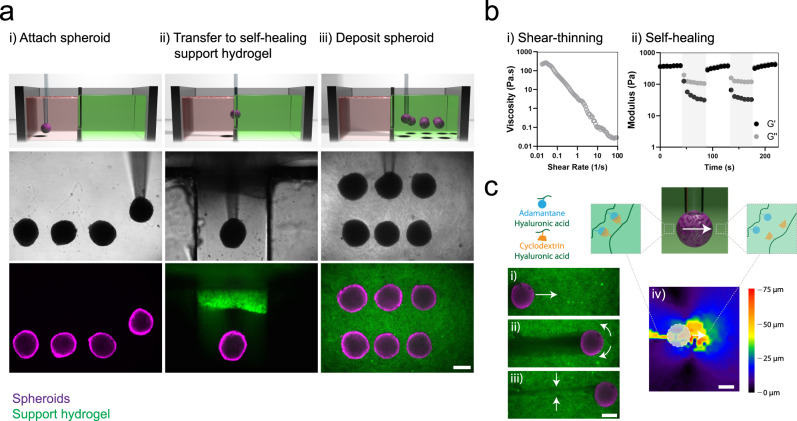
3D bioprinting spheroids in self-healing support hydrogels. **a** Schematic (top), brightfield images (middle), and fluorescent images (bottom) demonstrating, (i) MSC spheroid aspiration in a media reservoir, (ii) spheroid transfer into a self-healing support hydrogel (FITC-labeled), and (iii) spheroid deposition within the support hydrogel through removal of vacuum from the micropipette tip. Images are representative of *n* = 4 independent experiments. **b** Rheological characterization of a guest-host support hydrogel (3 wt%) demonstrating, (i) shear-thinning properties—decreased viscosity with continuously increasing shear rates (0–100 s^−1^) and (ii) self-healing properties—storage and loss modulus recovery (G′ & G″) through low (0.5% strain, 10 Hz) and high (shaded, 100% strain, 10 Hz) strain cycles. **c** Reversible interactions between guest (adamantane, blue) and host (β-cyclodextrin, orange) modified hyaluronic acid of the support hydrogel (containing FITC-microparticles) enable, (i–ii) local yielding of the support hydrogel under shear during spheroid translation, and (iii) rapid healing of the support hydrogel after spheroid translation. (iv) Displacement mapping of the support hydrogel (spheroid noted as dashed circle) demonstrating local motion of the hydrogel in front of and behind the spheroid during spheroid translation. Images are representative of *n* = 3 independent experiments. All scalebars 250 µm.

To enable the controlled patterning of spheroids, we developed a method for the bioprinting of spheroids within self-healing support hydrogels, which involves: (i) vacuum aspiration of a spheroid in a media reservoir, (ii) direct transfer of the spheroid into a support hydrogel due to its shear-thinning properties, and (iii) deposition of the spheroid at any location in the hydrogel with vacuum removal and self-healing of the hydrogel (Fig. 1a i–iii, Supplementary Movie 1). This process is enabled by the separation of the media reservoir containing the spheroids from a second reservoir containing the support hydrogel by a narrow channel for spheroid transport between the two reservoirs (Supplementary Fig. 1, Supplementary Movie 1). This approach avoids surface tension effects that can significantly reduce cell viability when transferring cells and spheroids between liquid and air interfaces^31^. The support hydrogel used is based on the assembly of hyaluronic acid (HA) modified with either adamantane (Ad) or β-cyclodextrin (CD) (Supplementary Fig. 2), resulting in shear-thinning and self-healing properties, as represented by a reduction in hydrogel viscosity with increasing shear rate and a recovery of storage modulus during cycles of high to low strain (Fig. 1b). This supramolecular hydrogel was developed previously for applications in the delivery of therapeutics to tissues and as a bioink for extrusion printing^32–34^.

Spheroids were translated from the media reservoir to the support gel at a constant speed of 400 µm/s, resulting in a per spheroid print time of ~40 s (12 mm total distance). This is comparable to other hydrogel-based spheroid printing methods reported at ~30 s/spheroid^31^. Higher speeds have been reported with the kenzan method (~10 s/spheroid), although this approach does involve mechanical disruption of spheroids. While recently developed spheroid printing methods such as one termed SWIFT possess higher scalability^35^, the single spheroid resolution that can be achieved with our approach is particularly advantageous for control of local tissue heterogeneity, which will find utility for in vitro models or organ-on-a-chip technologies. To visualize hydrogel motion during spheroid bioprinting, we embedded FITC-microparticles within the support hydrogel and used particle image velocimetry (PIV) analysis to track particle motion. Shear-thinning properties allowed the support hydrogel to be locally disrupted to accommodate spheroid translation (Fig. 1c i–ii, Supplementary Movie 2) and self-healing properties allowed the hydrogel to reassemble to hold the spheroid in place following the release of vacuum (Fig. 1c iii, Supplementary Movie 2). Quantitative mapping of relative particle motion indicated that the hydrogel yielded predominantly at the front of the translating spheroid with limited hydrogel movement further than ~200 µm from the spheroid (Fig. 1c iv, Supplementary Fig. 3). Due to inherent hydrogel properties, the bioprinting process could be repeated, allowing the deposition of multiple spheroids within the hydrogel at any location in 3D space not previously occupied (Fig. 1a). The position of previously printed spheroids must be considered to avoid disrupting already deposited spheroids (Supplementary Fig. 1d).

**Fig. 2:**
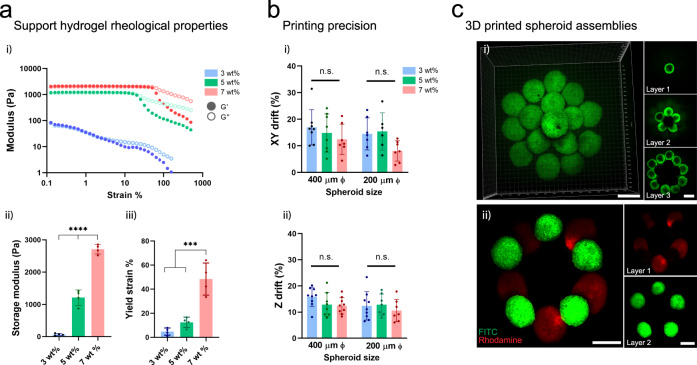
Support hydrogel rheological properties and 3D bioprinting precision. **a** (i) Shear-yielding in guest-host support hydrogels with increasing strain (0.1–500%, 10 Hz) at varied macromer concentrations (3, 5, 7 wt%) (storage modulus G′, closed circles; loss modulus G″, open circles). (ii) Storage modulus at low strain (0.5%) at varied macromer concentrations (*n* = 4 hydrogel samples, mean ± s.d, one-way ANOVA, 3 vs. 5 wt% *p* = 1.0 × 10^−5^, 5 vs. 7 wt% *p* = 1.0 × 10^−6^, 3 vs. 7 wt% *p* = 5.0 × 10^−9^). (iii) Yield point (strain at G′/G″ crossover) at varied macromer concentrations (*n* = 4 hydrogel samples, mean ± s.d, one-way ANOVA, 3 vs. 7 wt% *p* = 1.0 × 10^−4^, 5 vs. 7 wt% *p* = 4.6 × 10^−4^). **b** (i) Bioprinting precision in the XY plane (XY drift %) for 200 and 400 µm diameter spheroids (*n* = 8, 8, 7, 7, 6, 7 biologically independent samples (from left to right), mean ± s.d, one-way ANOVA). XY drift % = post-printing spheroid drift/spheroid diameter (see Supplementary Fig. 4a i for schematic of measurement). (ii) Bioprinting precision in the Z plane (Z drift %) for 200 and 400 µm diameter spheroids (*n* = 9, 8, 9, 9, 7, 7 biologically independent samples (from left to right), mean ± s.d, one-way ANOVA). Z drift % = post-printing spheroid drift/spheroid diameter (see Supplementary Fig. 4a ii for schematic of measurement). **c** 3D bioprinted MSC spheroids within a support hydrogel (3 wt% macromer concentration) into either (i) a multi-layer cone shaped geometry (FITC-labeled spheroids) or (ii) layered rings of distinct MSC spheroid populations (FITC- or rhodamine-labeled) within the support hydrogel. All scalebars 250 µm. All experiments are from a single MSC donor. (n.s. not significant, ***p < 0.001, ****p < 0.0001).

Having illustrated the ability to bioprint spheroids inside self-healing support hydrogels, we next explored how the hydrogel rheological properties influenced bioprinting precision. Increasing the polymer concentration from 3 to 7 wt% resulted in hydrogels with higher storage moduli that required higher strains to initiate yielding (Fig. 2a i–iii). To assess bioprinting precision, we developed assays where the XY and Z locations of a bioprinted spheroid is tracked during culture in the support hydrogel post-printing, which was performed for two spheroid sizes (200 and 400 µm Ø) and across polymer concentrations from 3 to 7 wt% (Supplementary Fig. 4a). For XY precision, we measured drift distances of ~8–15% of the spheroid diameter (or ~15–60 µm) for all polymer concentrations (Fig. 2b i, Supplementary Fig. 4b i). For Z precision, we measured similar drift distances of ~10–15% of the spheroid diameter (or ~25–60 µm) for all polymer concentrations (Fig. 2b ii, Supplementary Fig. 4b ii). These drift distances are likely caused by disruption of the gel by the translating micropipette after spheroid deposition, where the support gel heals rapidly but a displacement is introduced after moving the micropipette away (Supplementary Movie 1). This can also be visualized with fluorescent mapping of hydrogel displacement during spheroid translation (to the left of the spheroid) (Fig. 1c iv). Generally, these distances are small, which indicates high precision of bioprinted spheroids and supports the approach for the high resolution bioprinting of spheroids into patterns.

Next, we evaluated the viability of cells within bioprinted spheroids 24 h post-printing for all polymer concentrations (Supplementary Fig. 5). At lower polymer concentrations (3 and 5 wt%) high cell viabilities (~90–95%) were found, whereas a significant decrease in cell viability (~87%) was observed at the highest polymer concentration (7 wt%). Lower polymer concentrations likely reduce the impact of shear forces during spheroid translation, resulting in higher cell viabilities. Due to the high bioprinting precision across all polymer concentrations, and highest cell viabilities at low polymer concentrations, we utilized the 3 wt% hydrogels for all subsequent studies. To highlight the versatility of our bioprinting approach, we deposited multiple spheroids in close contact into a layered pyramid structure (Fig. 2c i) or as two distinct spheroid populations (pre-labelled with FITC and rhodamine cell masks) into layered rings (Fig. 2c ii). These results demonstrate that the shear-thinning and self-healing properties of support hydrogels allow spheroids to be freely moved within the hydrogel for patterning of multi-spheroid structures, without technically challenging intermediate layering and crosslinking steps. There are numerous shear-thinning and self-healing hydrogels that may be useful for this approach; however, these findings indicate that the hydrogel should be evaluated with respect to precision and cell viability prior to use^36^.

### 3D bioprinting high-cell density tissue models through directed spheroid fusion

**Fig. 3:**
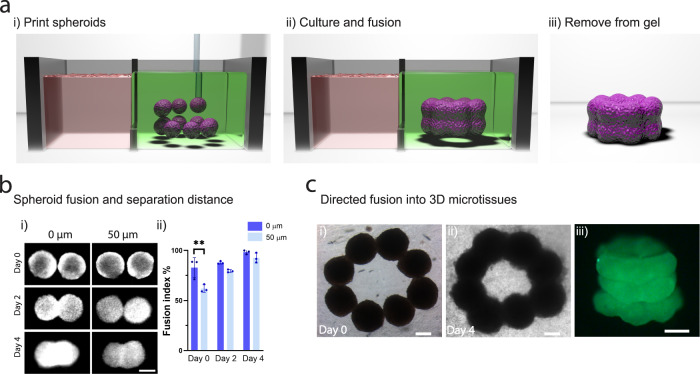
3D bioprinting microtissues through directed spheroid fusion. **a** (i) Schematic demonstrating the bioprinting of spheroids into rings within the support hydrogel, (ii) spheroid fusion and microtissue formation within the support hydrogel during culture, and (iii) removal of the microtissue via washing of the support hydrogel. **b** (i) Spheroid fusion over 4 days as a function of initial separation distance (0 or 50 µm). (ii) Fusion index (%) (see Supplementary Fig. 6a for schematic of measurement) over 4 days of culture (*n* = 3 biologically independent samples, mean ± s.d, one-way ANOVA, day 0 – 0 vs. 50 µm *p* = 0.0025). **c** (i–ii) Brightfield images of spheroid fusion into microtissue rings over 4 days of culture. (iii) 3D bioprinted microtissue tube composed of 3 layers of fused spheroids (after 4 days of culture and then removal from the support hydrogel). All scalebars 200 µm. All experiments are from a single MSC donor. (**p < 0.01).

Having established that we could 3D bioprint spheroids at high-resolution inside self-healing support hydrogels, we next explored if directed spheroid fusion could be used to fabricate multi-spheroid microtissues with controlled structures (Fig. 3a). When spheroids were bioprinted with direct contact, fusion was observed over four days of culture (Fig. 3b i–ii). In addition, when spheroids were bioprinted at a separation distance of 50 µm, comparable levels of fusion were observed (Fig. 3b ii). These distances are within the measured precision of our method, minimizing any concern that spheroids can be bioprinted with close enough proximity for spheroid fusion. Leveraging these properties, we next deposited eight spheroids into a ring pattern, which allowed continuous fusion into a microtissue ring after 4 days of culture (Fig. 3c i–ii). This process could be repeated with multiple layers of spheroids to form a microtissue tube (Fig. 3c iii). Importantly, due to the non-covalent and reversible nature of crosslinks in the support hydrogel, along with the soft hydrogel mechanical properties (G′ ~150 Pa), it was possible to gently pipette away the support hydrogel for removal from the fused microtissues (Fig. 3c iii).

When a secondary covalent network was introduced into the support hydrogel to induce more elastic behavior (Supplementary Fig. 6b i, ii), spheroid fusion was significantly reduced (Supplementary Fig. 6b iii). This highlights the importance of the biophysical properties of the support hydrogel in facilitating spheroid fusion. Spheroids display liquid-like behavior and undergo coalescence in a similar manner to liquid droplets^37^, which has been explained using the differential adhesion hypothesis where cells rearrange to maximize adhesive cadherin bonding and reduce free energy^38^. The viscous properties of the shear-thinning hydrogel investigated here allows cells to remodel their surrounding environment in protease-independent mechanisms with local hydrogel yielding at the spheroid-hydrogel interface^39^, which is reduced in more elastic networks. Such properties should certainly be considered when selecting the appropriate support hydrogel in this approach.

Together these results establish that self-healing hydrogels can be used to support spheroid assembly and fusion into larger 3D microtissues of prescribed structures. The system has a number of advantages when compared to traditional cells-in-gels bioprinting approaches. For example, a significant challenge in the field of biofabrication has been the development of bioinks that meet the rigorous criteria to be processed using extrusion and lithography approaches, while also supporting robust cell differentiation, proliferation, and migration^40^. Here, the biological considerations can be decoupled from the biofabrication process, allowing growth of multi-cellular structures of prescribed 3D patterns. Further, it is possible to fabricate models at organotypic cell densities at early culture times, which is a significant challenge in the bioprinting field, particularly for applications where high levels of cell-cell contact are central to function.

### 3D bioprinting cardiac microtissues for disease modelling applications

There are numerous applications that would benefit from the ability to bioprint high cell density and heterogeneous tissue models. For example, there has been great interest in the engineering of cardiac disease models for personalized medicine applications, including for the screening of drugs for efficacy and toxicity. Indeed, cardiovascular toxicity in therapeutic drug development represents the highest incidence of complications in late-stage clinical development^41^. Much can be learned from animal models; however, in vitro models are more controlled to address fundamental biological questions, can be used with a wide range of imaging and assessment techniques, and can be formulated from patient cells. This has guided the development of engineered human cardiac tissue models that provide controlled environments to study cardiac development and disease along with drug toxicities in vitro^42,43^. Many technologies are being developed, including self-assembled spheroids/organoids^6,44–46^, organ-on-a-chip^47,48^, and microtissue platforms^42,43,49^. In addition, to better mimic cardiac disease, pathological features are being introduced using hypoxia-induced apoptosis^6^, mechanical stress^50^, or varied cardiac cell ratios^51^. Recent examples have also demonstrated that the introduction of electrical barriers such as holes in healthy cardiac tissue or fibroblast dense regions disrupts action potential propagation^46,52^.

Despite significant advances in this area, engineering tissue models that accurately replicate the pathophysiology of cardiac disease remains a challenge. Nearly all cardiac diseases involve pathological fibrotic remodeling caused by CF proliferation and ECM deposition^53^. CFs themselves cannot generate action potentials, and excessive ECM production reduces cardiomyocyte-cardiomyocyte (CM) connectivity, leading to reduced tissue compliance and electrical synchronization^53^. Focal cardiac fibrosis that arises post-MI is a major clinical burden that often leads to a cascade of pathological remodeling and may result in heart failure^53^. Engineered tissue models that can replicate aspects of these pathological features and allow the study of repair mechanisms, could provide a valuable tool for developing therapeutic interventions^54^.

**Fig. 4:**
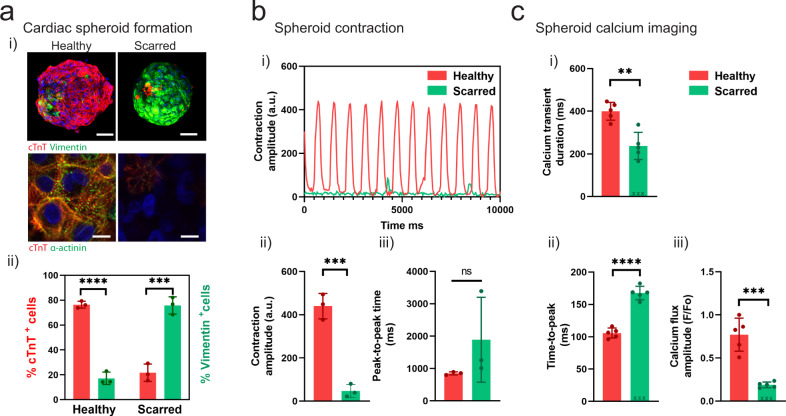
Fabrication of cardiac spheroids for disease modeling applications. **a** (i) Development of healthy and scarred spheroids through mixing iPSC-derived cardiomyocytes (iPSC-CMs) with primary adult cardiac fibroblasts (CFs) at defined ratios of cell numbers (4:1 for healthy; 1:4 for scarred). Top: Images (cardiac troponin-T (cTnT) (red; iPSC-CMs); vimentin (green; CFs)) taken 3 days after cell seeding. Scalebar 50 µm. Bottom: Immunofluorescence staining for alpha-actinin (green; sarcomeres) and cTnT (red; iPSC-CMs) in healthy and scarred spheroids at 3 days. Scalebar 10 µm. (ii) Quantification of cellular composition through staining for cTnT (iPSC-CMs) and vimentin (CFs) (*n* = 3 biologically independent samples, mean ± s.d, two-sided student *t* test, healthy - cTnT^+^ vs. Vimentin^+^ *p* = 5.6 × 10^−5^, scarred - cTnT^+^ vs. Vimentin^+^ *p* = 6.7 × 10^−4^). **b** (i) Contraction profiles, (ii) contraction amplitude (a.u. absolute units), and (iii) peak-to-peak time (ms) of healthy and scarred cardiac spheroids at 3 days (*n* = 3 biologically independent samples, mean ± s.d, two-sided student *t* test, (ii) *p* = 5.0 × 10^−4^). **c** Quantification of calcium activation parameters from calcium mapping experiments in healthy and scarred spheroids at 3 days, including (i) calcium transient duration (ms), (ii) time-to-peak (ms), and (iii) calcium flux amplitude (F/Fo) (*n* = 5 biologically independent samples, mean ± s.d, two-sided student *t* test, (i) *p* = 0.0014, (ii) *p* = 5.0 × 10^−6^, (iii) *p* = 2.0 × 10^−4^). Note for *n* = 3 scarred spheroids a significant calcium activation peak was not observed, and these samples were not included in the statistical analysis and are denoted by x mark on the graph. All experiments are from a single iPSC-CM donor (donor A). (n.s. not significant, **p < 0.01, *** *p* < 0.001, **** *p* < 0.0001).

To address this need, we designed a reductionist model of focal cardiac fibrosis using our spheroid bioprinting technology. To mimic healthy and fibrotic cardiac tissue, we fabricated spheroids through the mixing of iPSC-derived cardiomyocytes (iPSC-CMs) and CFs at varied ratios (4:1 iPSC-CMs to CFs for “healthy”; 1:4 iPSC-CMs to CFs for “scarred”) (Fig. 4a i), which was validated via immunofluorescence staining for cardiac troponin-T (cTnT) (CM marker) and vimentin (CF marker) (Fig. 4a ii). iPSC-CMs are a common source of CMs for use in cardiac spheroid studies (Supplementary Table 1). Immunofluorescence staining for alpha-actinin also demonstrated robust sarcomere formation in healthy spheroids, with limited staining in scarred spheroids (Fig. 4a i). Next, we performed contraction recordings and used open-source musclemotion software to determine the contraction amplitude (which correlates with the force of contraction) and the peak-to-peak time (beating rate or rhythm)^55^. Healthy spheroids had a significantly increased contraction amplitude when compared to scarred controls at 3 days, as well as a trend toward a faster peak-to-peak time of ~850 ms (~70 bpm) (Fig. 4b). Optical mapping of intracellular Ca^2+^ showed robust and synchronized Ca^2+^ flux in healthy spheroids (Supplementary Movie 3), whereas lower levels and only dispersed and sporadic islands of Ca^2+^ flux were observed in scarred spheroids (Supplementary Movie 3). Next, we quantified key parameters of the cardiac cell cycle including the calcium transient duration (CTD) (ms), time-to-peak (ms), and the calcium flux amplitude (F/Fo) (Fig. 4c). The CTD and calcium flux amplitude were significantly higher in healthy spheroids (Fig. 4c i,iii), and the time-to-peak was significantly lower (i.e., faster depolarization) in healthy spheroids (Fig. 4c ii). Together, these results demonstrate that variations in the CM:CF ratio in cardiac spheroids allow the creation of models of healthy and scarred cardiac tissue.

**Fig. 5:**
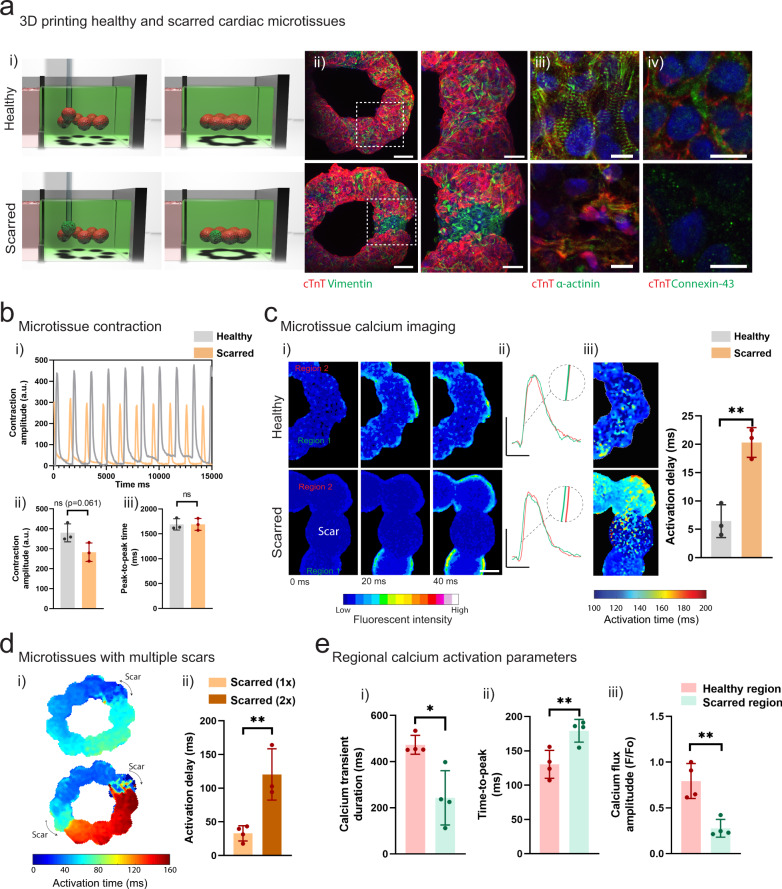
3D bioprinting cardiac microtissues for disease modeling applications. **a** (i) Schematic of 3D bioprinting of healthy and scarred cardiac microtissue rings and (ii) immunofluorescence staining for cTnT and vimentin in healthy and scarred cardiac microtissues after 5 days of fusion within the support hydrogel. Scalebar 100 µm (insets 50 µm). (iii) Immunofluorescence staining for alpha-actinin (green; sarcomeres) and cTnT (red; iPSC-CMs) and (iv) connexin-43 (green; gap junctions) and cTnT (red; iPSC-CMs), in healthy and scarred regions of microtissues after 5 days of fusion within the support hydrogel. Scalebar 10 µm. **b** (i) Contraction profiles of healthy and scarred cardiac microtissues following removal from the support hydrogel after 5 days of culture. (ii) Contraction amplitude (a.u. absolute units) and (iii) Peak-to-peak time (ms) at 5 days (*n* = 3 biologically independent samples, mean ± s.d, two-sided student *t* test, (ii) *p* = 0.061). **c** (i) Calcium mapping in healthy and scarred cardiac microtissues after 5 days culture, each image represents a 20 ms frame. Scalebar 100 µm. (ii) Representative calcium traces from regions 1 and 2 (see methods) in healthy and scarred cardiac microtissues. Scalebars 0.5 ΔF/F_o_ (y), 500 ms (x). (iii) Activation maps of healthy and scarred cardiac microtissues, and activation delay (ms) (difference in activation time (ms) between regions 1 and 2) in healthy and scarred cardiac microtissues (*n* = 3 biologically independent samples, mean ± s.d, two-sided student *t* test, *p* = 0.0035). **d** (i) Activation maps of scarred cardiac microtissues with 1 or 2 scars after 5 days of culture and (ii) quantification of activation delay (ms) (*n* = 3-4 biologically independent samples, mean ± s.d, two-sided student *t* test, *p* = 0.0066). **e** Regional quantification of (i) calcium transient duration (ms), (ii) time-to-peak (ms), and (iii) calcium flux amplitude (F/Fo), in healthy and scarred regions of microtissues (scarred 1x) after 5 days of culture (*n* = 4 biologically independent samples, mean ± s.d, two-sided student *t* test, (i) *p* = 0.010, (ii) *p* = 0.0098, (iii) *p* = 0.0029). Note full calcium transient duration, time-to-peak, and activation maps can be found in Supplementary Fig. 8. All experiments from a single iPSC-CM donor (donor A). (n.s. not significant, *p < 0.05, **p < 0.01).

To model focal cardiac fibrosis, we used our bioprinting technology to create heterogenous rings of cardiac tissue through the directed fusion of healthy and scarred spheroids. A ring structure was used to mimic the tissue level electrophysiological properties of a heart chamber, including the possibility of re-entrant arrhythmias. To model healthy myocardium, we bioprinted rings of eight healthy spheroids in direct contact, whereas models of scarred myocardium were bioprinted with rings of seven healthy spheroids and one scarred spheroid (Fig. 5a i). After 5 days of culture, dual staining for cTnT and vimentin confirmed fusion between adjacent spheroids, and regions of high fibroblast density were observed in scarred microtissues (Fig. 5a ii). Live-dead staining demonstrated high cell-viability after 5 days of fusion (Supplementary Fig. 7b), and the ratio of iPSC-CMs to CFs remained constant over the culture period (Supplementary Fig. 7c). Robust sarcomere staining was also observed in healthy regions of the microtissues, with lower levels observed in scarred regions (Fig. 5a iii). In addition, staining for connexin-43 at cell boundaries indicated gap-junction formation in healthy regions of the microtissues with lower levels of staining found in scarred regions (Fig. 5a iv). The microtissue rings also contracted in a continuous and synchronized fashion, which could be observed following removal from the support hydrogel (Fig. 5b i, Supplementary Movie 4). Quantification of microtissue contraction revealed trends toward reduced contraction amplitudes in scarred microtissues when compared to healthy controls (Fig. 5b ii–iii), mimicking reductions in global contractility that arise following MI.

Next, we used optical Ca^2+^ mapping to assess how fibrotic scars influence electrophysiological properties of microtissues. Healthy microtissues demonstrated synchronized calcium activation propagation across the entire ring, indicating functional electrical coupling between fused spheroids (Fig. 5c i, Supplementary Movie 5). In contrast, wave-like propagation was observed in scarred microtissues (Fig. 5c i, Supplementary Movie 5). Examination of sequential frames from the fluorescent recordings demonstrated limited calcium flux in the scar, and delayed activation between healthy regions on both sides of the scar (Fig. 5c i, Supplementary Movie 5). To visualize activation delays, we plotted calcium activation events in the two healthy regions directly adjacent to the scar, which demonstrated reduced overlap when compared to measurements across the same positioned spheroids in healthy controls (Fig. 5c ii). In addition, activation maps also confirmed increased delays across scarred regions (Fig. 5c iii), which could be quantified as the average activation delay (difference in activation time before and after scar) and was significantly higher than in healthy controls (Fig. 5c iii).

To further demonstrate the modularity of our bioprinting approach for disease modelling applications, we printed microtissues containing multiple scars to explore if this would exacerbate electrical desynchronization in healthy tissue. To do this, we printed eight healthy spheroids and two scarred spheroids (at opposing ends of the ring) followed by fusion for 5 days in the support hydrogel (scarred 2×), and compared this to single scar controls containing nine healthy spheroids and one scarred spheroid (scarred 1×). Optical mapping of calcium flux demonstrated significantly increased activation delays in microtissues containing two scars when compared to single scar controls (Fig. 5d i, ii). We did not observe re-entry circuits forming in microtissues containing single or multiple scars. Finally, we quantified local activation parameters in the healthy and scarred regions of our microtissues (scarred 1×), demonstrating reduced CTD and calcium flux amplitude, along with a slower time-to-peak in scarred regions (Fig. 5e i–iii, Supplementary Fig. 8). This data confirms lower levels of electrical activation within the scarred regions of the microtissues, which leads to microtissue level activation delays (reduced synchronization). Together, this demonstrates that printed scars disrupt electrophysiological synchronization in engineered cardiac microtissues, which provides a platform to explore how heterocellular interactions can influence electrophysiological properties in cardiac disease states.

Here, we developed a versatile microtissue platform for focal cardiac fibrosis modelling that mimics the two main structural features that arise following scarring (reduced contractile output and electrical synchronization). We chose a relatively simple microtissue design (planar ring, one or two scars) as in vitro models should be designed using a reductionist approach that allows monitoring of variables of interest^56^. We also demonstrated that the system is modular and can be easily adopted to model how multiple pockets of scarred tissue influence electrical synchronization. The ability to create healthy and diseased regions in a single microtissue also offers the opportunity to probe borderzone interactions and study aspects of cardiac repair in vitro.

In contrast to our approach, traditional extrusion-based bioprinting approaches can be challenging to implement for cardiac models. Functional cardiac muscle requires high levels of cell-cell contact to support rapid action potential propagation and synchronized contraction. Fibrillar hydrogels such as collagen and fibrin are compatible with cardiac tissue engineering as the cells can rapidly contract the matrix to form high cell density tissues^49^; however, these softer hydrogels are challenging to process using biofabrication technologies^40^. The organotypic cell densities that can be achieved using our self-assembled spheroid approach also supports high levels of CM-CM contact, which is analogous to healthy cardiac tissue. It should be noted that spheroid-based microtissues developed in our system lack the anisotropic tissue alignment present in mature myocardium and do not include vascular networks that are highly abundant in native myocardium; however, future efforts could advance the platform with additional control over spheroid anisotropy and the incorporation of endothelial cells, as has been previously described for cardiac spheroids^45^.

### Evaluating the behavior of cardiac microtissues in response to miRNA therapeutics

Having established a 3D bioprinted cardiac fibrosis model, we next looked to utilize the system to study microRNA (miRNA) therapeutics for cardiac repair. Following cell death within the ischemic environment after MI, cardiac tissue has limited capacity for self-repair in the adult, particularly due to the non-proliferative nature of CMs. Recent pre-clinical evidence indicates that Hippo pathway inhibiting miRNAs can stimulate CM proliferation^34,57,58^. For example, the delivery of Hippo inhibiting miR302 b/c mimics stimulates cardiomyocyte proliferation and cardiac repair following MI in mice^34^. However, in a more recent porcine MI model, prolonged expression of Hippo inhibiting miRNA-199a through viral transfection resulted in sudden cardiac failure in 70% of the treated pigs^59^. This was attributed to excessive cardiomyocyte dedifferentiation and proliferation that produced islands of non-contractile tissue and fatal arrhythmias. Our bioprinted cardiac microtissues present an opportunity to evaluate how the extent of miR302 b/c induced Hippo inhibition (treatment duration) influences cardiomyocyte proliferation and cardiac repair (e.g., contractility, calcium propagation).

**Fig. 6:**
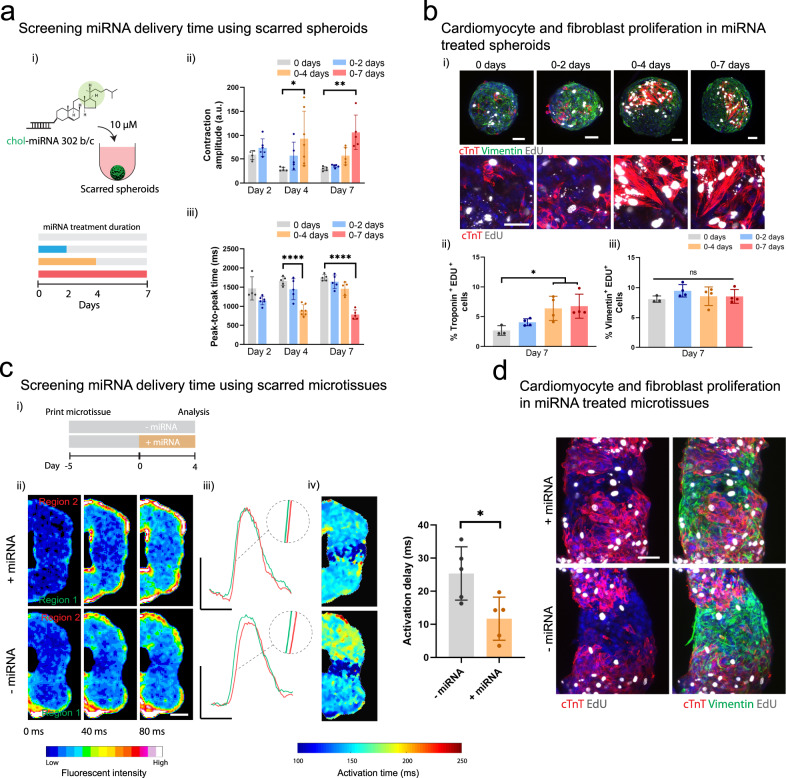
Evaluating miRNA on the behavior of cardiac microtissues. **a** (i) Schematic of cholesterol modified miR302 (chol-miRNA 302 b/c) delivery to scarred cardiac spheroids for 0, 0–2, 0–4, and 0–7 days. (ii) Contraction amplitude (a.u) and (iii) peak-to-peak time (ms) within scarred spheroids measured after 2, 4, and 7 days for each treatment period (*n* = 4, 6, 5, 5, 7, 5, 5, 5, 5 biologically independent samples (from left to right), mean ± s.d, one-way ANOVA, (ii) day 4: 0 vs. 0–4 days treatment *p* = 0.014, (ii) day 7: 0 vs. 0–7 days treatment *p* = 0.0045, (iii) day 4: 0 vs. 0–4 days treatment *p* = 1.6 × 10^−7^, (iii) day 7: 0 vs. 0–7 days treatment *p* = 4.1 × 10^−9^). **b** (i) Immunofluorescence staining for cTnT (red; iPSC-CMs), vimentin (green; cardiac fibroblasts), and EdU (proliferation marker) in scarred spheroids at day 7 for each treatment condition. Top panel scalebar 50 µm, and bottom panel scalebar 40 µm. (ii) Quantification of cardiomyocyte proliferation (EdU^+^ and cTnT^+^) and (iii) fibroblast proliferation (EdU^+^ and Vimentin^+^) at day 7 for each treatment condition. (*n* = 3, 4, 4, 4 biologically independent samples (from left to right), mean ± s.d, one-way ANOVA, 0 vs. 0–4 days treatment *p* = 0.044, 0 vs. 0–7 days treatment *p* = 0.026). Scalebar 50 µm. **c** (i) Experimental outline where scarred microtissues are bioprinted in the support hydrogel as previously described, followed by 4 days treatment with miR302, and analysis compared to non-treated controls. (ii) Calcium mapping in scarred cardiac microtissues at 9 days (5 days culture in support hydrogel; (+) and (−) 4 days miRNA treatment) each frame 40 ms. Scalebar 100 µm. (iii) Representative calcium traces from regions 1 and 2 in treated and non-treated scarred cardiac microtissues. Scalebars 0.5 ΔF/F_o_ (y), 500 ms (x). (iv) Activation time maps across scarred regions for treated and non-treated scarred cardiac microtissues, and quantification of activation delay (ms) (*n* = 5 biologically independent samples, mean ± s.d, two-sided student *t* test, *p* = 0.019). **d** Immunofluorescence staining for cTnT (iPSC-CMs), vimentin (CFs), and EdU (proliferation) in scarred cardiac microtissues at day 9 for treated and non-treated scarred cardiac microtissues. Scalebar 50 µm. All experiments from a single iPSC-CM donor (donor B). (n.s. not significant, *p < 0.05, **p < 0.01, **** *p* < 0.0001).

First, we screened a range of miRNA treatment durations (0, 0–2, 0–4, and 0–7 days) using our scarred cardiac spheroids (Fig. 6a i). To assess the transient effects of miRNA treatment, we performed sequential contraction imaging at 2, 4, and 7 days. Significant increases in contraction amplitude were observed with constant treatment for 4 and 7 days when compared to untreated controls at the same timepoint (Fig. 6a ii). Notably, contraction values increased approximately threefold for these conditions and approached ~20% of the contraction amplitudes present in healthy spheroids. The speed of contraction also increased significantly when compared to controls (~2× faster) when miRNA was still present in the media (0–4 days treatment at 4 days, 0–7 days treatment at 7 days) (Fig. 6a iii). We also assessed how miRNA treatment influenced the contractile properties of healthy spheroids (Supplementary Fig. 9a i). No significant changes in contraction amplitude were observed in healthy spheroids (Supplementary Fig. 9a ii), with an increase in contraction speed at earlier timepoints only (0–2 days treatment at 2 days) (Supplementary Fig. 9a iii).

To determine if miRNA treatment promoted CM and CF proliferation in scarred spheroids, we performed EdU labelling (a proliferation marker) along with co-staining for cTnT and vimentin staining at 7 days (Fig. 6b i). Islands of cTnT^+^EdU^+^ were present, suggesting proliferation and clonal expansion (Fig. 6b i), particularly with longer treatment durations (0–4 days, 0–7 days) (Fig. 6b ii). No increases in the number of Vimentin^+^ Edu^+^ cells were observed, indicating that miRNA treatment was primarily influencing CM proliferation (Fig. 6b iii). Similar trends were also observed in healthy spheroids, with increases in the number of cTnT^+^ and Edu^+^ cells observed for all treatment conditions when compared to controls (Supplementary Fig. 9b i–ii) and limited effects on CF proliferation (Supplementary Fig. 9b iii).

Having established that miR302b/c mimics stimulate CM proliferation and increase the functional properties of scarred spheroids, we next assessed if these improvements could translate into tissue level repair using our scarred microtissue model. To do this, scarred microtissues were bioprinted as previously described, followed by treatment with miRNA for 4 days (Fig. 6c i). We chose 4 days of treatment, as further improvements in contraction or CM proliferation were not observed with 7 days of continuous treatment. After 4 days of treatment, we assessed tissue-level electrophysiological properties through calcium mapping (Fig. 6c ii). Further corroborating the improvements found with single spheroids, miRNA treatment significantly reduced activation delays present in scarred microtissues when compared to untreated controls (Fig. 6c iii, iv). In addition, miRNA treatment increased the CTD and calcium flux amplitude, and significantly reduced the time-to-peak in scarred regions of the microtissues. (Supplementary Fig. 10 i–iii), which indicates improved electrical activation. Finally, histological staining at the scar interface allowed us to visualize tissue remodeling in response to miRNA treatment (Fig. 6d).

Engineered cardiac tissues are being widely used as models to study drug toxicities and efficacy in vitro^6,43–45,60^. Here, we developed a fibrosis model that enabled the study of therapeutics for cardiac repair, including with functional outcomes to assess changes in contraction and electrophysiological properties. The introduction of scars adjacent to healthy tissue allowed us to study tissue-level electrophysiological properties and our results demonstrate that these fibrotic regions are responsive to therapeutics. In our model, miR302 b/c mimics enhanced iPSC-CM proliferation, resulting in enhanced contractility in scarred spheroids and improved electrophysiological integration. miRNA treatment did not enhance CF proliferation which is an important consideration when designing miRNA therapeutics for cardiac repair and corroborates previous 2D cultures of iPSC-CMs and CFs with miRNA treatment^61^.

iPSC cardiomyocytes are a promising platform to model cardiac development and disease in vitro, and can be used for personalized drug screening applications^62,63^. Although iPSC-CMs exhibit many characteristics of primary human cardiomyocytes, their functional properties are somewhat immature and mimic aspects of fetal rather than adult cardiomyocytes^62^. This is an important consideration for disease modelling and drug screening applications. The structure and function of iPSC-CMs can be matured using mechanical and electrical stimulation^42,49^, prolonged culture periods^64,65^, and also co-culture in the presence of CFs or endothelial cells^66^. For example, co-culture of CFs with iPSC-CMs has been shown to increase cardiomyocyte maturity, resulting in improved electrophysiological maturity in similar cardiac spheroid models^66^. In our study, we observed robust sarcomere assembly (alpha-actinin) in both spheroids and microtissues (Figs. 4a i, 5a iii). We observed positive staining for connexin-43 at cell boundaries in healthy spheroids indicating gap-junction formation at early timepoints (3 days after seeding), with weaker staining in scarred spheroids (Supplementary Fig. 7 a). In addition, increased staining for connexin-43 at the cell boundaries was observed in printed microtissues after 5 days of fusion in the support hydrogel (Fig. 5 a iv). Together these results indicate our microtissue system can support early cardiac maturation, which is likely supported by the high levels of cell-cell contact and presence of adult CFs.

Our findings highlight how 3D bioprinting technology can be used to design personalized in vitro disease models that provide comparable functional outputs to pre-clinical animal models, while keeping investigations simple, minimizing costs, and in formats that support a wide variety of imaging and assessment techniques. Beyond the cardiac model we have presented here, we believe the precision of our bioprinting method could enable advances in the fabrication of assembloids, where organoids from different tissues or tissue regions are fused in a controlled environment to study tissue development and maturation^67,68^.

Advances in bioprinting technologies have enabled the development of in vitro models and implantable constructs that better mimic the complexity of native tissues and organs. Many of these technologies rely on processing cell suspensions embedded in hydrogels, making it challenging to engineer tissues with native tissue-like cell densities and heterogeneity, and resulting functions. Here, we developed a process that enables the 3D bioprinting of high cell density tissue models, with precise control over microtissue structure and local heterogeneity. This is enabled by the application of self-healing hydrogels that support the bioassembly and fusion of bioprinted spheroids in 3D space to form continuous cell-dense tissue models. We demonstrate the potential of the approach by bioprinting cardiac microtissue disease models that recapitulate pathological scarring features that arise post-MI, and using readouts for cardiac function (contraction, electrophysiological synchronization) we were able to probe miRNA therapeutics for repair. The approach is highly generalizable and could be implemented with a wide range of spheroid and organoid systems, which opens up many opportunities in 3D bioprinting of precision models to mimic diseases and for drug screening.

## Methods

### Hydrogel preparation

HA macromers (adamantane (Ad)-HA, cyclodextrin (CD)-HA) were synthesized using previously described protocols^32,69^. First, the tetrabutylammonium salt of sodium hyaluronate (Lifecore, 64 kDa) (HA-TBA) was prepared using Dowex 50 W proton exchange resin. Ad was coupled to the tetrabutylammonium salt of HA (HA-TBA) to obtain Ad-HA through 4-dimethylaminopyridine (DMAP)/ditert-butyl dicarbonate (BOC_2_O)-mediated esterification. For CD-HA, aminated β-CD was coupled to HA-TBA via amidation in the presence of benzotriazole-1-yl-oxy-tris-(dimethylamino)-phosphonium hexafluorophosphate (BOP). Products were dialyzed, frozen, and lyophilized. Next, ^1^H NMR (DMX 360 MHz, Bruker; Topspin for data analysis) was used to determine that Ad-HA had ~18.9% of HA repeat units modified with Ad (Supplementary Fig. 2a) and CD-HA contained ~15.4% of HA repeat units modified with β-CD (Supplementary Fig. 2b) AdNorHA was synthesized by simultaneous coupling of Ad and 5-norbornene-2-carboxylic acid (Nor) to HA-TBA through DMAP/BOC_2_O mediated esterification for 20 h at 45 °C^70^. To form support hydrogels, Ad-HA and CD-HA macromers were separately dissolved in PBS and then mixed at a 1:1 Ad:CD ratio and final polymer concentrations of 3, 5, and 7 wt%. To introduce secondary covalent crosslinking into the hydrogel, Ad-HA was replaced with AdNorHA and supplemented with 1 mM DL-dithiothreitol (DTT) and 0.05 wt% photoinitiator (lithium phenyl-2,4,6-trimethylbenzoylphosphinate (LAP), Colorado Photopolymer Solutions (Boulder, CO)). Crosslinking was performed with the application of visible light (2 mW cm^−2^ intensity, 400–500 nm wavelength, 3 min exposure). For a more detailed description of all hydrogel synthesis protocols the readers are redirected to the following protocol paper^71^.

### Cell culture and spheroid formation

Human MSCs (hMSCs) were isolated from fresh unprocessed bone marrow from human donors (Lonza, 1M-125). Briefly, bone marrow was diluted in alpha-modified essential medium (α-MEM, 10% FBS, 1% penicillin/streptomycin, 5 ng ml^−1^ basic fibroblast growth factor) (1:10 ratio) and plated on tissue culture plastic at 37 °C/5% CO_2_. hMSCs were cultured until 80% confluency of the colonies, followed by storage in liquid nitrogen (95% FBS, 5% DMSO). Next, hMSCs were expanded in standard growth media (α-MEM, 10% FBS, 1% penicillin/ streptomycin, 5 ng ml^−1^ basic fibroblast growth factor) for one passage before spheroid formation. hMSC spheroids were prepared by centrifugation in ultra-low attachment 96-well round-bottom plates (Corning 7007, 500 G). Two spheroid sizes were prepared by adding either 5000 or 10,000 hMSCs/well, which resulted in average spheroid diameters of ~200 µm and ~400 µm (Supplementary Fig. 1c). For visualization, hMSC spheroids were labelled with Cell Tracker^™^ fluorescent probes (Green-CMFDA, Red CMPTX, 5 µM).

Human iPSC-CMs were purchased from Fujifilm Cellular Dynamics (iCell 01434 (Donor A) and 11713 (Donor B), purity >97%). Human CFs were purchased from Promocell and expanded in α-MEM (10% FBS, 1% penicillin/streptomycin, 5 ng ml^−1^ basic fibroblast growth factor) at 37 °C/5% CO_2_ until 80% confluency. Cardiac spheroids were formed by mixing iPSC-CMs and CFs at different ratios (4:1 for “healthy” and 1:4 for “scarred”, 5000 cells total) in ultra-low attachment 96-well round-bottom plates. For the first 48 h, cardiac spheroids were cultured in iCell plating medium (Cellular Dynamics) to optimize post-thawing survival of the iPSC-CMs. After 48 h, cardiac spheroids were maintained in iCell maintenance media (Cellular Dynamics). Approximately 24 h after thawing, the iPSC-CM and CF co-cultures had aggregated into a single spheroid with synchronized contractions observed after 24–48 h. For all further experiments, cardiac spheroids were used at 96 h after thawing.

### Rheological characterization

The viscoelastic properties of the support hydrogels were characterized using shear rheometry (cone and plate geometry with (0.995°) cone angle, 27 µm gap, TA Instruments, AR2000) at 25 °C. To assess shear-thinning properties, viscosity was measured as a function of shear rate (0–50 s^−1^). Shear-recovery was assessed by applying low (0.5%) and high (100%) strains periodically at 10 Hz. Strain yielding behavior was assessed using strain sweeps (0–500%, 10 Hz).

### 3D bioprinting setup

Custom molds for 3D bioprinting were drawn using computer-aided design (CAD) software (Solidworks) and then 3D bioprinted from Accura SL 5530 (Proto Labs). PDMS was then cast against the molds and bonded to a glass coverslip (Supplementary Fig. 1a). The mold system consisted of two reservoirs (200 µl volume) connected by a narrow channel (750 µm width) (Supplementary Fig. 1a). The first reservoir was filled with cell culture media and spheroids and the second reservoir was filled with the support hydrogel (Supplementary Fig. 1a). The connecting channel was designed to prevent flow between the two reservoirs. Next, the PDMS mold containing spheroids and support hydrogel was placed in a live cell incubation chamber (37°, 5% CO_2_) and a spinning disk confocal (Nikon TE-2000) was used to visualize spheroids during bioprinting (Supplementary Fig. 1b)_._ To bioprint spheroids in the support hydrogel, a micromanipulator with XYZ control (Transferman^®^, Eppendorf) and containing custom pulled micropipettes (Ø 100 µm) was used to vacuum aspirate the spheroids and transfer them into the support hydrogel via the connecting channel (400 µm/s), and then deposit them with vacuum removal (Supplementary Fig. 1a,b, Supplementary Movie 1).

To visualize the support hydrogel during bioprinting, green-fluorescent polystyrene beads were added at a 1:200 dilution (nominal diameter ~1 µm, emission/excitation 441/485 nm, Fluoresbrite^®^). Spheroids were translated through the hydrogel at a constant speed (400 µm/s) and microparticle motion was tracked using fluorescent videos (20 HZ) (Supplementary Movie 2). To measure bead displacements, image frames were isolated from the videos every 50 frames, and the relative bead motion between frames was used to produce vector and displacements maps using the PIV plugin (Image-J) (Supplementary Fig. 3).

### 3D bioprinting accuracy measurements

To quantify bioprinting precision in the XY plane, a partially transparent target was superimposed on the bottom glass surface of the bioprinting mold (Supplementary Fig. 4a i) and a spheroid was bioprinted directly above the target. Next, the bioprinting micropipette was removed, and the post-printing drift was measured after 5 min by measuring the distance between the center of the spheroid and the target. To quantify bioprinting precision in the Z plane, a spheroid was deposited in the support hydrogel and the z-position was monitored over 24 h using brightfield videos (Supplementary Fig. 4a ii).

### Live/dead staining

For viability analysis, spheroids were stained using a Live/Dead cell-viability assay (Invitrogen) according to the manufacturer’s instructions (2 µM calcein AM and 4 µM EthD-1). Viability was quantified from confocal stacks (250 µm thick) acquired using a Leica SP5 II confocal microscope. The live cell area was reported as the ratio of calcein-AM stained area to the total spheroid area (sum of calcein-AM stained area and ethidium homodimer-1 stained area). Note, live cell area was used as a metric instead of per cell viability due to challenges in quantifying per cell viability in dense spheroids with the abundance of the cytosolic calcein signal making it difficult to visualize cell boundaries. As the calcein is a cytosolic signal and Ethd-1 is nuclear localized, the live cell area is an approximate heuristic for viability.

### 3D bioprinting MSC spheroid microtissues and spheroid fusion measurements

To 3D bioprint microtissues, eight hMSC spheroids were bioprinted into the support hydrogel in a circular ring with direct contact between adjacent spheroids (Supplementary Fig. 1d). Molds containing bioprinted spheroids in the support hydrogel were cultured at 37 °C/5% CO_2_ and the media reservoir was refreshed every two days. For all hMSC spheroid experiments, α-MEM (10% FBS, 1% penicillin/streptomycin, 5 ng ml^−1^ basic fibroblast growth factor) was used. To track spheroid fusion dynamics, hMSC spheroids were labelled with Cell Tracker^™^ fluorescent probes (Green-CMFDA). Spheroid fusion was tracked using brightfield and fluorescent imaging, and robust fusion was observed after 4 days of culture in the support hydrogel. To remove microtissues from the support hydrogel, excess gel around the microtissues was gently pipetted away from the reservoir and the microtissues were removed from the support hydrogel through gentle aspiration using a micropipette tip, followed by transfer back into the media reservoir. The spheroid fusion index was calculated as described (Supplementary Fig. 6).

### 3D bioprinting cardiac microtissues and contraction imaging

To 3D bioprint healthy cardiac microtissues, eight healthy spheroids were bioprinted into the support hydrogel in a circular ring as described above. Scarred microtissues were bioprinted by including one scarred spheroid and seven healthy spheroids into a circular ring (Fig. 5a-c). In a separate experiment, to compare the effect of having multiple scars within a single microtissue, ten total spheroids were bioprinted in a ring that contained two scarred spheroids at opposite ends of the ring (Fig. 5d, e). For all cardiac spheroid experiments, samples were maintained in iCell maintenance media (Cellular Dynamics) at 37°/5% CO_2_. After 5 days of culture and spheroid fusion, the bioprinted cardiac rings were removed from the support hydrogels using aspiration (after gently removing excess support gel) and transferred back into the media reservoir. A thin layer of agarose (3 wt%) was added to the bottom surface to prevent attachment of the microtissues to the coverslip. Microtissue contraction was recorded at 37°/5% CO_2_ in iCell maintenance media using a Nikon TE2000 (4× lens, 20 HZ). The software package musclemotion was used for all contraction analysis^55^. The contraction amplitude (absolute unit, a.u) is an optical measure of contraction and is widely used for cardiac spheroid studies and correlates with the force of contraction, which has been validated across single adult CMs, iPSC-CMs, cardiac spheroids, and engineered heart tissues^55^. The contraction amplitude (a.u) is automatically normalized to the pixel density of the video recording, and a consistent pixel density was used across all recordings^55^.

### Calcium imaging and analysis

To assess calcium activation, microtissues were treated with intracellular calcium indicator (Cal 520, AAT Bioquest, 4 µM) for 90 min. Pluronic (0.04%) was included to enhance cellular uptake and all staining solutions were prepared in iCell maintenance media. Following calcium loading, the staining solution was replaced with maintenance media and fluorescence images of calcium concentration were acquired with a Nikon Eclipse TE2000U microscope fitted with a spinning disk confocal (CSU-10b, Solamere Technologies), a CCD camera (Photometric Cool-Snap HQ^2^, BioVision, Exton PA) (4 × 4 binning to 174 × 130 pixels), 488 nm excitation laser (Prairie SFC), and a Nikon 4X Plan Apo objective (N.A. = 0.2). Exposure time was set to 2 ms, and images were streamed at 50 Hz frame rate for at least 1000 frames using metamorph software.

The open-source software package ElectoMap was used to perform quantitative measurements on the acquired fluorescent calcium videos^72^. Activation maps were produced using the isochronal module using a spatial filter (2 pixels, sigma 1.5), and the activation measure was defined as the depolarization midpoint (the time taken for the calcium signal to reach 50% of its peak value during a single upstroke). Activation delays were then calculated by measuring differences in local activation time across the microtissues using the isochronal module. For example, in scarred microtissues the activation delay was calculated by measuring the difference in activation time in the two healthy regions (regions 1 & 2 in Fig. 5c i) directly adjacent to the scarred region. In healthy microtissues, the activation time was averaged in six evenly spaced regions across the microtissues, and the activation delay was calculated by measuring the average activation delay across each of these regions. The CTD maps were produced using the action potential duration model and measured from time of maximum upstroke velocity to 50% repolarization. Time-to-peak maps were produced using the time-to-peak module and defined as the time for the signal to reach peak calcium fluorescent intensity. For all calcium imaging mapping analysis, the background area around the microtissues was removed by tracing the outline of the microtissue in image-j. All calcium traces presented represent the ensemble averaged signal and were produced using ElectroMap^72^.

### Immunofluorescent staining and analysis

Microtissues were washed 3× times with PBS and then fixed overnight in 10% formalin at 4 °C. Following 3× washes with PBS, fixed microtissues were incubated for 4 h in permeabilization solution containing 0.2% Triton X-100 in PBS supplemented with 2% BSA, 320 mM sucrose and 6 mM magnesium chloride. Before immunostaining, microtissues were blocked in PBS containing 2% BSA for 2 h. Next, microtissues were stained with primary antibodies for cardiac troponin-t (cTnT) (Thermofisher MA5-12960, 1:200) and vimentin (Thermofisher MA5-16409; 1:200) to visualize cardiomyocytes and fibroblasts, respectively. To visualize sarcomeres, samples were stained with anti-sarcomeric alpha actinin antibody (abcam 137346; 1:200). To visualize gap junctions, samples were stained with connexin-43 (Thermofisher 71-0700; 1:200). Primary antibodies were diluted in PBS containing 3% BSA and hydrogels were stained at 4 °C for 72 h. Next, the secondary antibodies Alexa Fluor-488/594/647 IgG H&L (1:200; Abcam ab150077, ab150116, ab150079) were added at 4 °C for 72 h. Finally, samples were washed 3× in PBS followed by DAPI staining (1:2000; Invitrogen D1306, in PBS) for 30 min at room temperature. A Leica SP5 confocal was used to acquire z-stack images.

### miRNA treatments and proliferation analysis

Cholesterol modified miR-302b/c was purchased from GE Dharmacon. Their sequences are as follows.

Mus musculus miR-302b (miR-302b):

- 5′ -ACUUUAACAUGGGAAUGCUUUCU-3′ (guide)
- 3′ -GAUGAUUUUGUACCUUCGUGAAU-chol-5′ (passenger)

Mus musculus miR-302c (miR-302c):

- 5′ -GCUUUAACAUGGGGUUACCUGC-3′ (guide)
- 3′ -GGUGACUUUGUACCUUCGUGAA-chol-5′ (passenger)

For all treatments, miR-302b/c mimics were diluted in iCell maintenance media at 10 µM (each) and media was refreshed every 48 h. To assess cardiomyocyte and fibroblast proliferation during miRNA treatment, a Click-iT^®^ EdU Protocol (C10337 Thermofisher) was used. In this assay, the modified thymidine analog EdU (5-ethynyl-2′-deoxyuridine, a nucleoside analog of thymidine) is incorporated into newly synthesized DNA and fluorescently labeled with Alexa Fluor 488 dye. During miRNA treatment of cardiac microtissues, 5 µM EdU labeling solution was added to the culture media along with the miR-302b/c mimics. Then following fixing and immunofluorescence staining described above, EdU incorporated nuclei were visualized through a click reaction with Alexa Fluor 488 azide. Note for EdU staining with Alexa Fluor 488, the secondary antibodies Alexa Fluor-594/647 IgG H&L (1:200; Abcam ab150116, ab150079) were used to label the cTnT and vimentin primary antibodies. A Leica SP5 confocal was used to acquire z-stack images (~300 µm thick, 3 µm slices) and the number of EdU^+^/cTnT^+^ and EdU^+^/Vimentin^+^ cells were manually counted using the cell-counter plugin in image-J to quantify iPSC-CM and CF proliferation. The percentage of proliferating cells were then normalized to total cell number by quantifying the total number of DAPI stained nuclei.

### Statistical analysis

GraphPad Prism 8 software was used for all statistical analyses, and Microsoft excel was used for data handling. Statistical comparisons between two experimental groups were performed using two-tailed Student’s *t* tests and comparisons among more groups were performed using a one-way ANOVA with Tukey post hoc testing. Exact *p* values are provided in figure legends. Sample distribution was assumed normal with equal variance.

### Reporting summary

Further information on research design is available in the Nature Research Reporting Summary linked to this article.

## Supplementary information

Supplementary Information

Description of Additional Supplementary Files

Supplementary Movie 1

Supplementary Movie 2

Supplementary Movie 3

Supplementary Movie 4

Supplementary Movie 5

Reporting Summary

## Data Availability

All data supporting the results in this study are available within the article and its supplementary information or from the corresponding author upon reasonable request. A reporting summary for this Article is available as a Supplementary Information file. Source data are provided with this paper.

## Source data

Source Data
