# Supplementary material for: 3D bioprinting of high cell-density heterogeneous tissue models through spheroid fusion within self-healing hydrogels
Source: Nat Commun. 2021 Feb 2;12:753. doi: 10.1038/s41467-021-21029-2 (PMC7854667; doi:10.1038/s41467-021-21029-2)
Supplement: Supplementary file 1 — Supplementary Information [file 41467_2021_21029_MOESM1_ESM.pdf]

Supplementary material for

3D bioprinting of high cell-density heterogeneous tissue models through spheroid fusion within self-healing hydrogels

Andrew C. Daly[1], Matthew D. Davidson[1], Jason A. Burdick[1]*

[1]Department of Bioengineering, University of Pennsylvania, Philadelphia, PA, USA.

* Correspondence should be addressed to J.A.B. (email: burdick2@seas.upenn.edu)

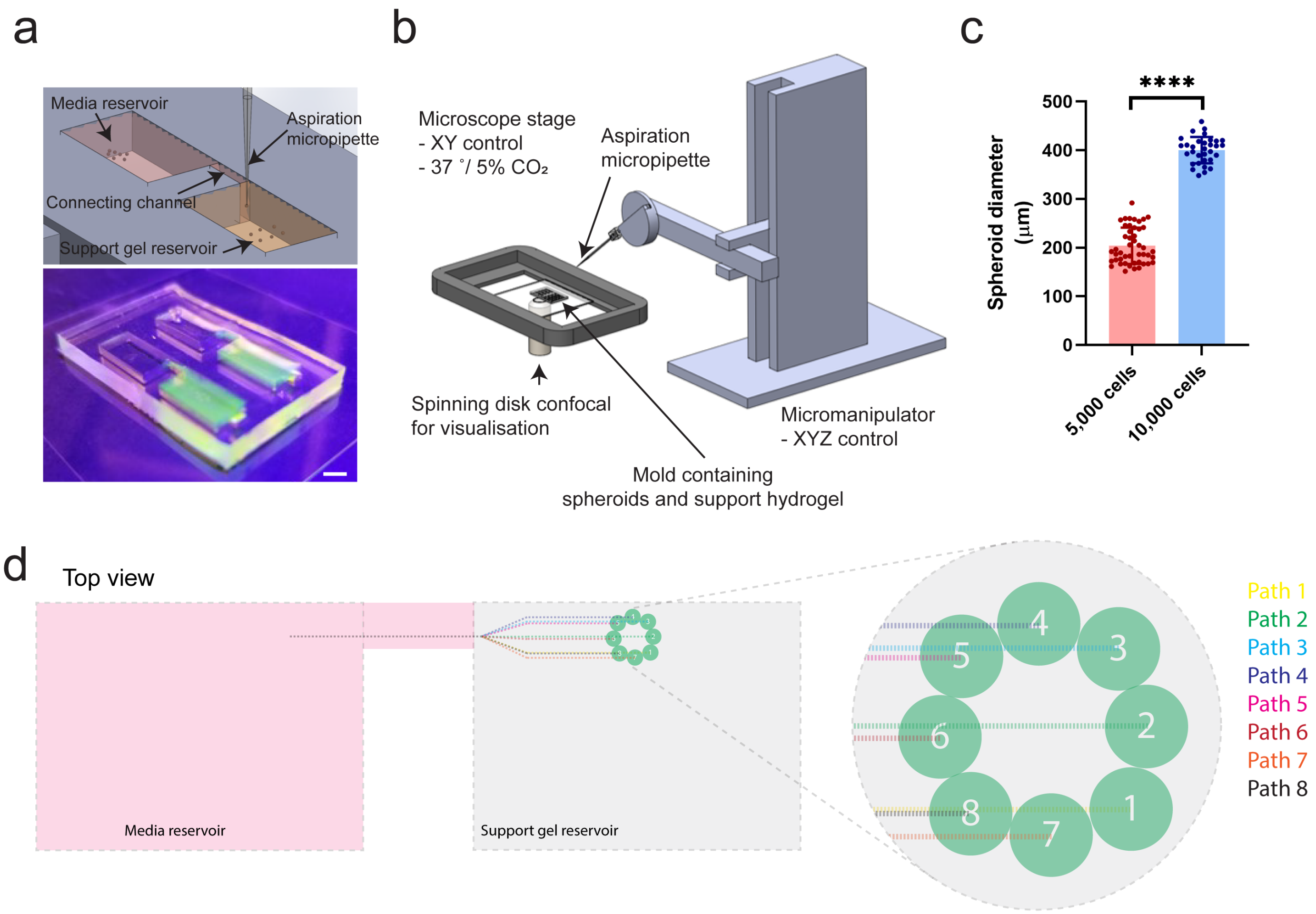

Supplementary Figure 1: 3D bioprinting approach and spheroid formation. (a) Schematic and image of the 3D bioprinting molds used to create a media reservoir (contains the formed spheroids) and a support gel reservoir (contains shear-thinning hydrogel) connected with a narrow channel, scale bar 7 mm. **(b)** Schematic of the 3D bioprinting setup composed of a robotic micromanipulator (XYZ spatial control) with a micropipette for spheroid aspiration, a microscope stage (XY spatial control) with an environmental chamber (37°C, 5% CO_2), and a spinning disk confocal for live brightfield and fluorescent imaging during bioprinting. **(c)** Average spheroid diameter (µm) 24 hours after seeding of either 5,000 or 10,000 human MSCs in ultra-low attachment 96 well round bottom plates. (n=47, 32 biologically independent samples, mean ± s.d, two-sided student t-test, $p<1.0\times10^{-15}$). **(d)** Schematic demonstrating the print-paths used to move single spheroids from the media reservoir to the support gel reservoir to create a microtissue ring (top view). All experiments are from a single MSC donor. (**** p<0.0001).

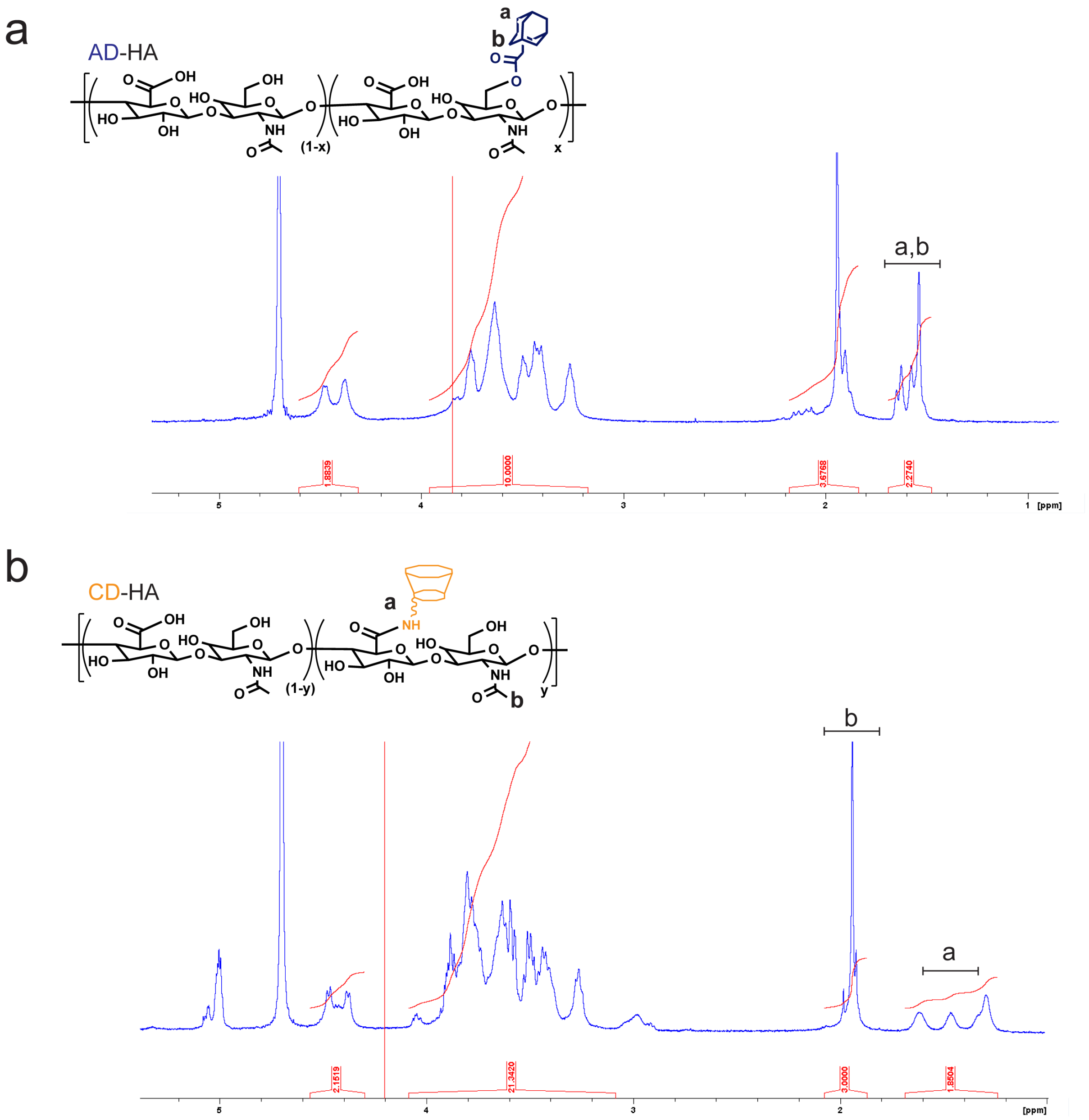

Supplementary Figure 2: Characterization of synthesized HA polymers. (a) ^{1}H NMR spectrum of adamantane modified hyaluronic acid (Ad-HA); the degree of adamantane functionalization (~18.9 %) relative to HA disaccharides (δ = 3.1 – 4.0, 10 H) is determined by integration of adamantane ethyl multiplet (δ = 1.5 - 1.7, 12 H). **(b)** ^{1}H NMR spectrum of β-cyclodextrin-modified hyaluronic acid (CD-HA); the degree of β-cyclodextrin functionalization (~15.4 %) relative to the HA methyl singlet (δ = 1.9 – 2.1, 3 H) is determined by integration of the hexane linker (δ = 1.2 - 1.7, 12 H).

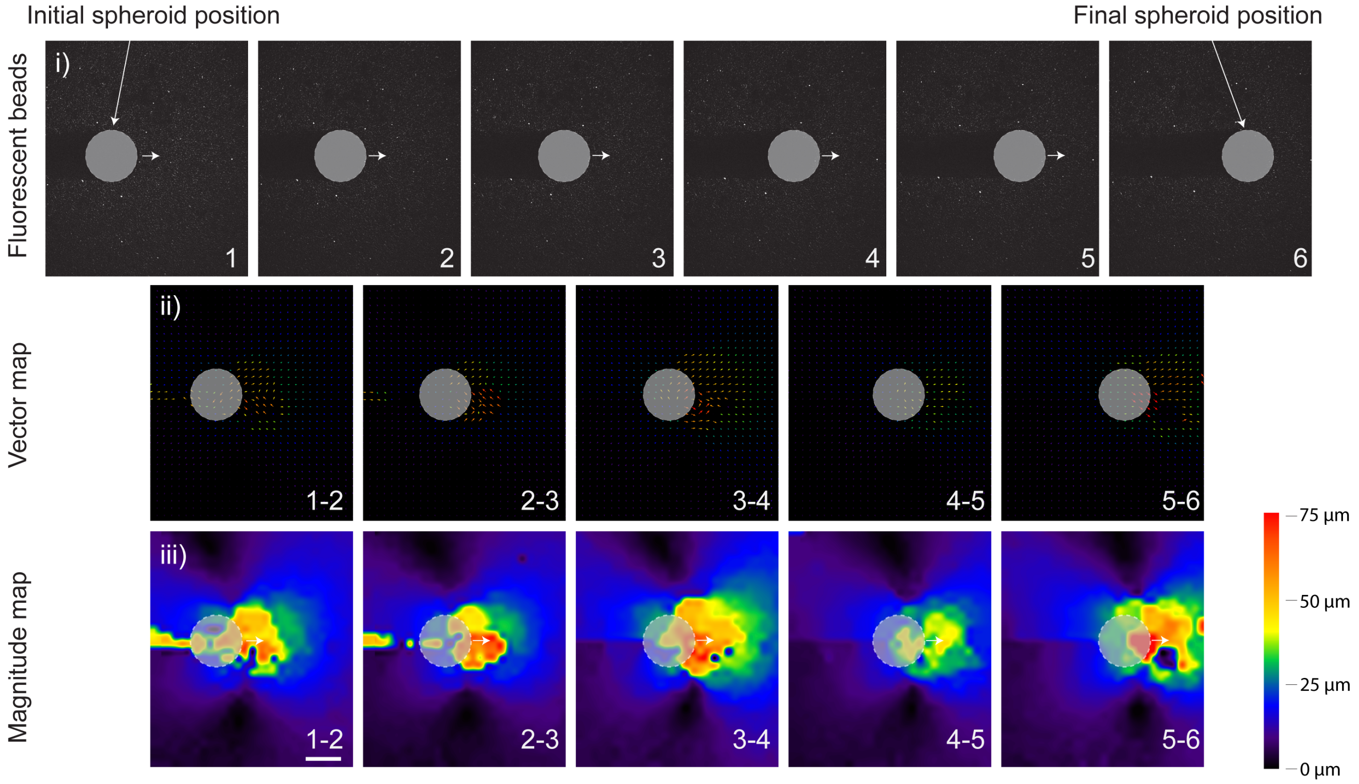

Supplementary Figure 3: Tracking support hydrogel motion during spheroid bioprinting. (i) Sequential images showing the motion of fluorescent beads within the support hydrogel during spheroid translation from left to right (1µm Ø fluorescent beads (white), 1:200 dilution). Particle image velocimetry (ii) vector plots and (iii) magnitude plots demonstrating relative bead motion between frames presented in the top panel, e.g. 1-2 represents the bead motion between images 1 and 2. Scalebar 200 µm. Images are representative of n=3 biologically independent samples.

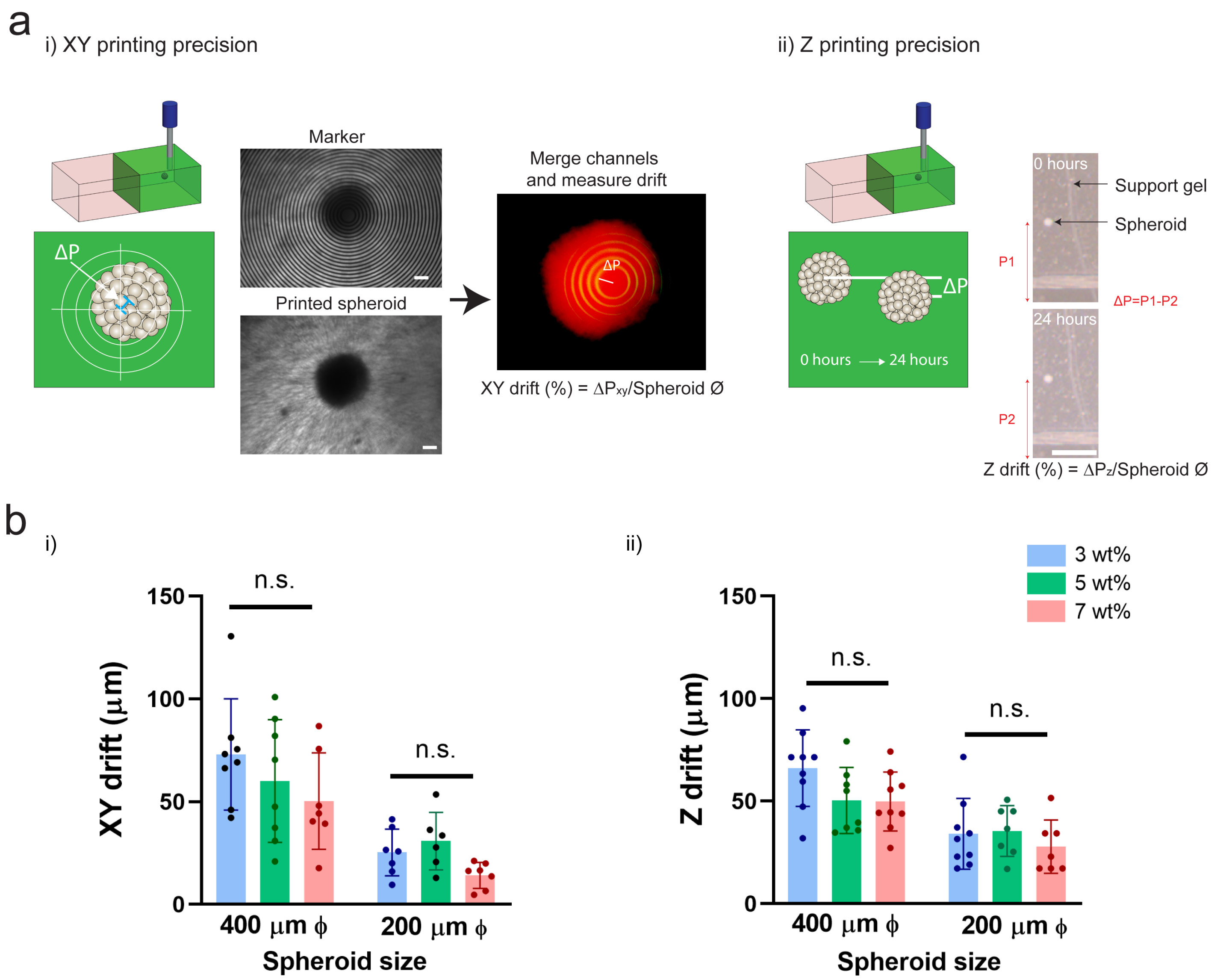

Supplementary Figure 4: 3D bioprinting precision measurements. (a) XY bioprinting precision method where a spheroid is deposited directly above a crosshatch marker and the post-printing spheroid drift (5 minutes after removing micropipette) is measured from the center of the spheroid. Scalebar 100µm. XY drift % = ΔP_{xy}/spheroid diameter. (b) Z bioprinting precision method where a bioprinted spheroid's z position is tracked in the support hydrogel over 24 hours. Scalebar 1mm. Z drift % = ΔP_{z}/spheroid diameter. (b) (i) Bioprinting precision in the XY plane (XY drift distance µm) for 200 and 400 µm diameter spheroids (n=8, 8, 7, 7, 6, 7 biologically independent samples (from left to right), mean ± s.d, one-way ANOVA). (ii) Bioprinting precision in the Z plane (Z drift distance µm) for 200 and 400 µm diameter spheroids (n=9, 8, 9, 9, 7, 7 biologically independent samples (from left to right), mean ± s.d, one-way ANOVA). All experiments are from a single MSC donor. (n.s. not significant).

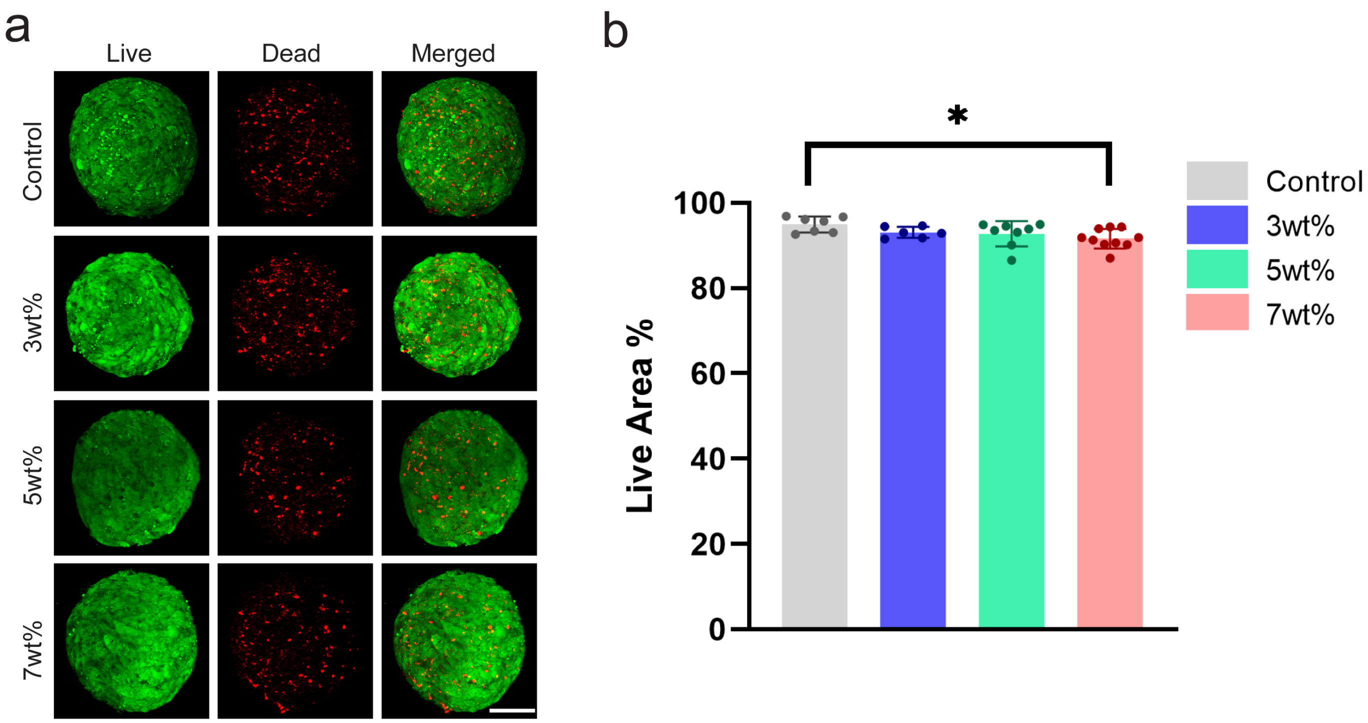

Supplementary Figure 5: Post-printing cell viability. (a) Spheroid live/dead staining in 3, 5, and 7 wt% support hydrogels compared to non-printed controls 24 hours post-printing (note: control spheroids were added to the media reservoir, but not bioprinted into a support hydrogel). Scalebar 100 μm. **(b)** Quantification of spheroid live area for each condition. (n=7, 6, 8, 10 biologically independent samples (from left to right), mean ± s.d, one-way ANOVA, control vs. 7wt% p=0.028). All experiments are from a single MSC donor. (*p<0.05).

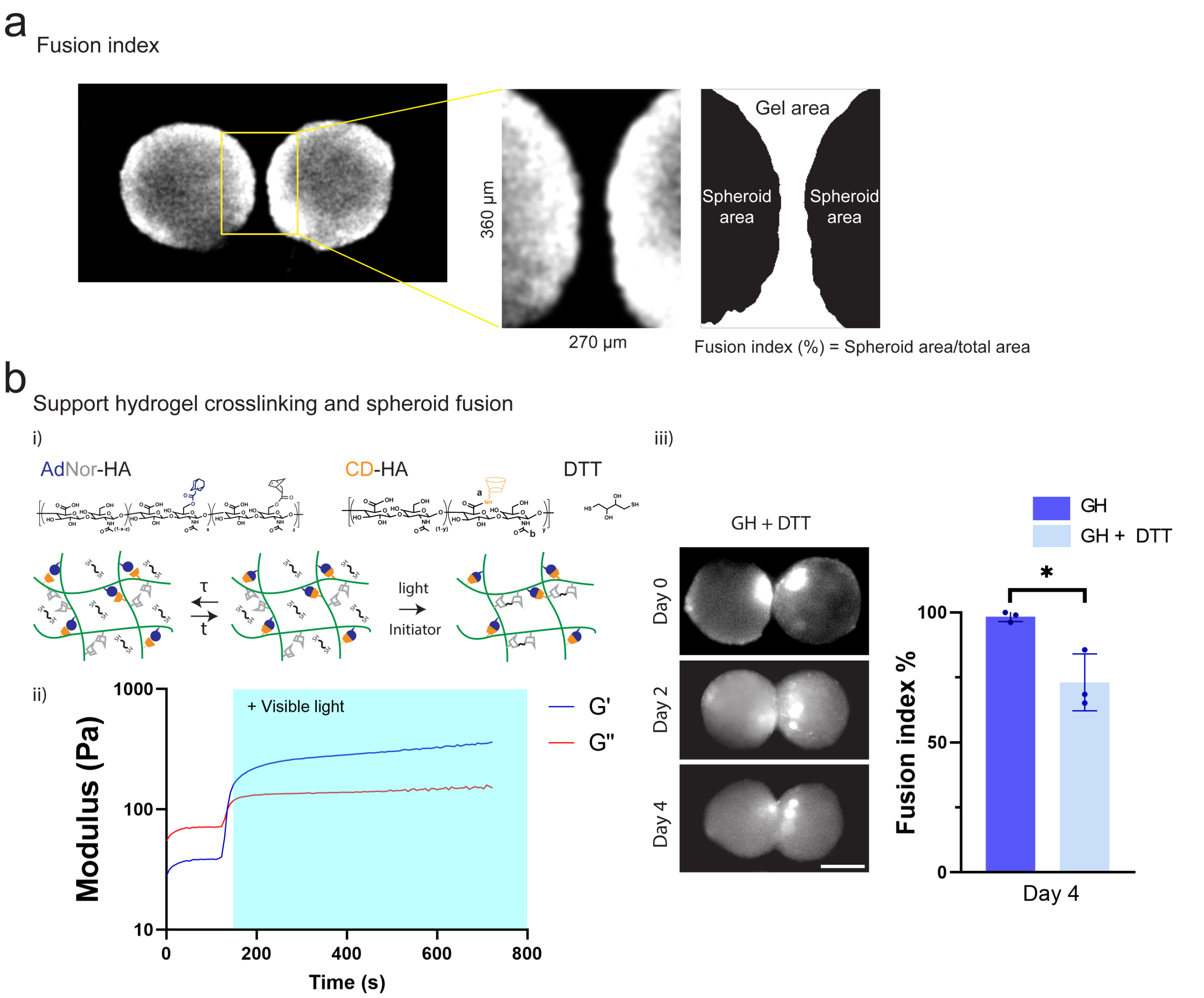

Supplementary Figure 6: Spheroid fusion dynamics. (a) Spheroid fusion measurements where the spheroid area (fluorescently labelled) and gel area (non-fluorescently labelled) between two adjacent spheroids (360 µm X 270 µm box) is quantified and used to determine the fusion index. Fusion index % = Spheroid area/ total area. Scalebar 200 µm. **(b)** (i) Secondary crosslinking of the guest-host support hydrogel through a thiol-ene reaction between norbornene groups and di-thiol crosslinker (DTT, non-degradable) in the presence of visible light. (ii) Rheological characterization of support hydrogel in response to secondary stabilization, time sweep (1 Hz, 1% strain). This indicates that the support hydrogel initially displays viscous behaviour, with a transition towards more elastic properties (increase in G') after thiol-ene crosslinking of norbornene groups with DTT (1mM DTT, 0.05% LAP photoinitiator, 2 mW cm^{-2} intensity 400-500 nm wavelength)**.** (iii) Spheroid fusion over 4 days with and without secondary crosslinking in the presence of DTT. (n=3 biologically independent samples, mean ± s.d, two-sided student t-test, p=0.017). Scalebar 200 µm. All experiments are from a single MSC donor. (*p<0.05).

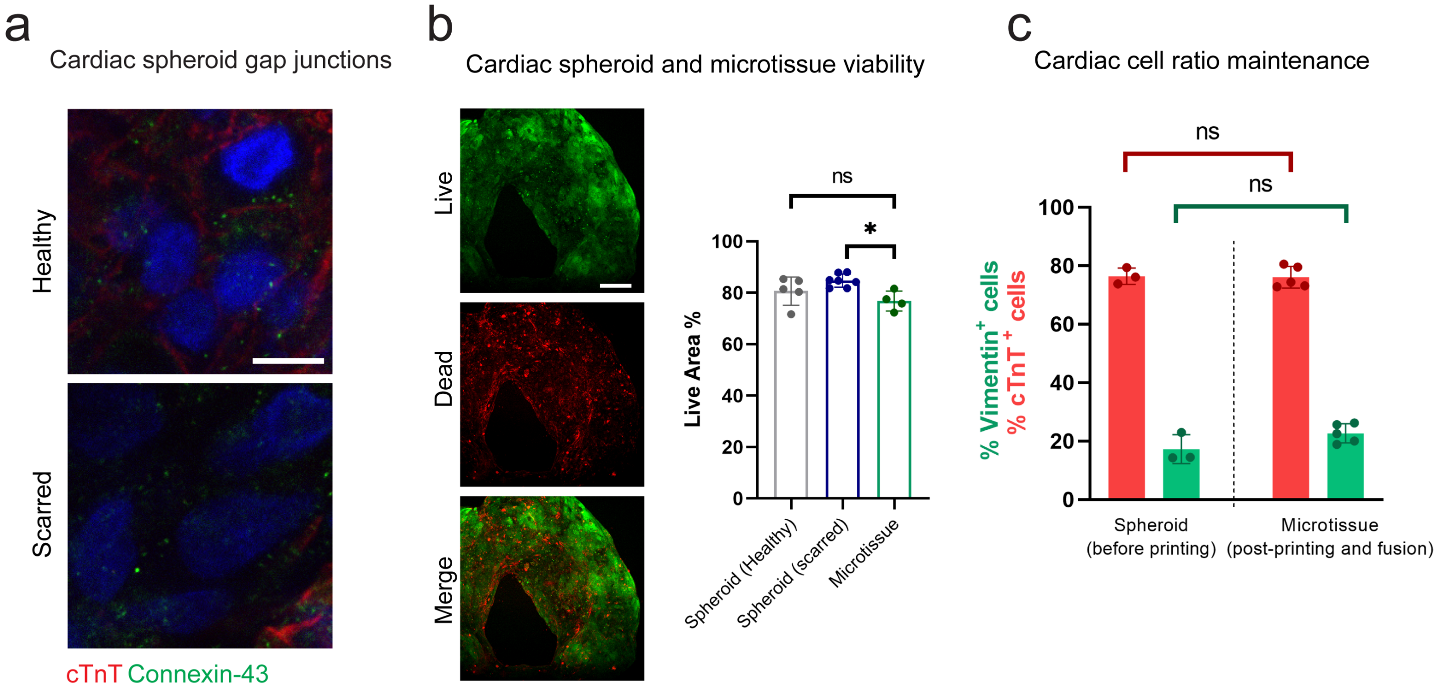

Supplementary Figure 7: Cardiac spheroid and microtissue properties during culture: (a) Immunofluorescence staining for connexin-43 (green; gap junctions) and cardiac troponin-T (cTnT) (red; iPSC-CMs) in healthy and scarred spheroids at 3 days (pre-printing). Scalebar 10 µm. Images are representative of n=2 (healthy spheroid) and n=3 (scarred spheroid) biologically independent samples. (**b)** Live/dead staining of cardiac microtissues 5 days after printing. Scalebar 100 µm. Quantification of live area (%) in cardiac microtissues 5 days post-printing, compared to cardiac spheroids (healthy and scarred) at 3 days (pre-printing), (n=5, 7, 4 biologically independent samples (from left to right), mean ± s.d, one-way ANOVA, p=0.018). **(c)** Quantification of cellular composition through staining for cTnT (iPSC-CMs) and vimentin (CFs) in healthy spheroids at 3 days (pre-printing) and microtissues at 5 days post-printing (n=3, 3, 5, 5 biologically independent samples, mean ± s.d, one-way ANOVA). All experiments are from a single iPSC-CM donor (donor A). (n.s. not significant, *p<0.05).

Regional calcium activation parameters in scarred microtissues

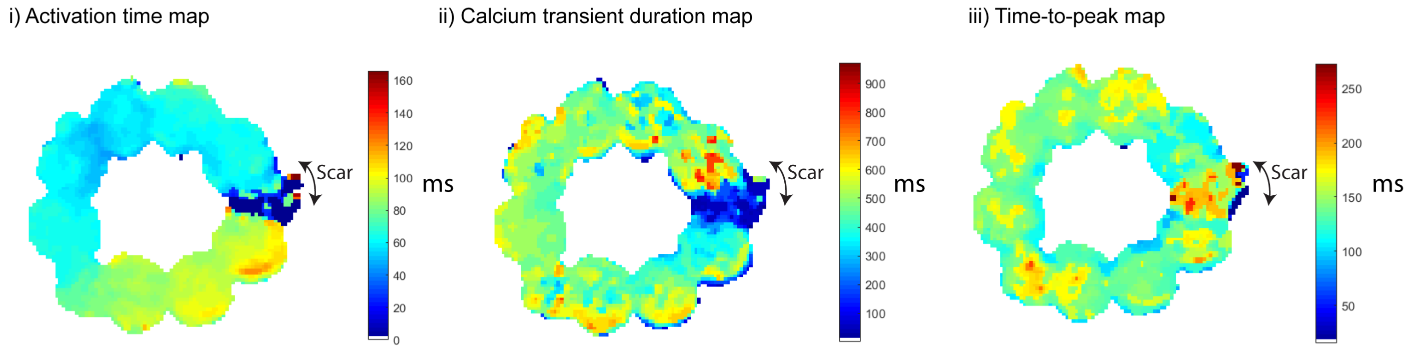

Supplementary Figure 8: Regional calcium activation parameters in scarred microtissues. (i) Activation map (ms), (ii) calcium transient duration map (ms), and (iii) time-to-peak map (ms), in scarred cardiac microtissues at 5 days post-printing (single scar). Activation time is defined as the time taken for the calcium signal to reach 50% of its peak value during a single upstroke, calcium transient duration is measured at 50% of peak value, and time-to-peak is defined as the time to reach the peak calcium intensity during an upstroke. Images are representative maps from n=4 biologically independent samples and the mean values for each parameter in healthy and scarred regions have been quantified for comparison in Fig. 5d, e. All experiments are from a single iPSC-CM donor (donor A).

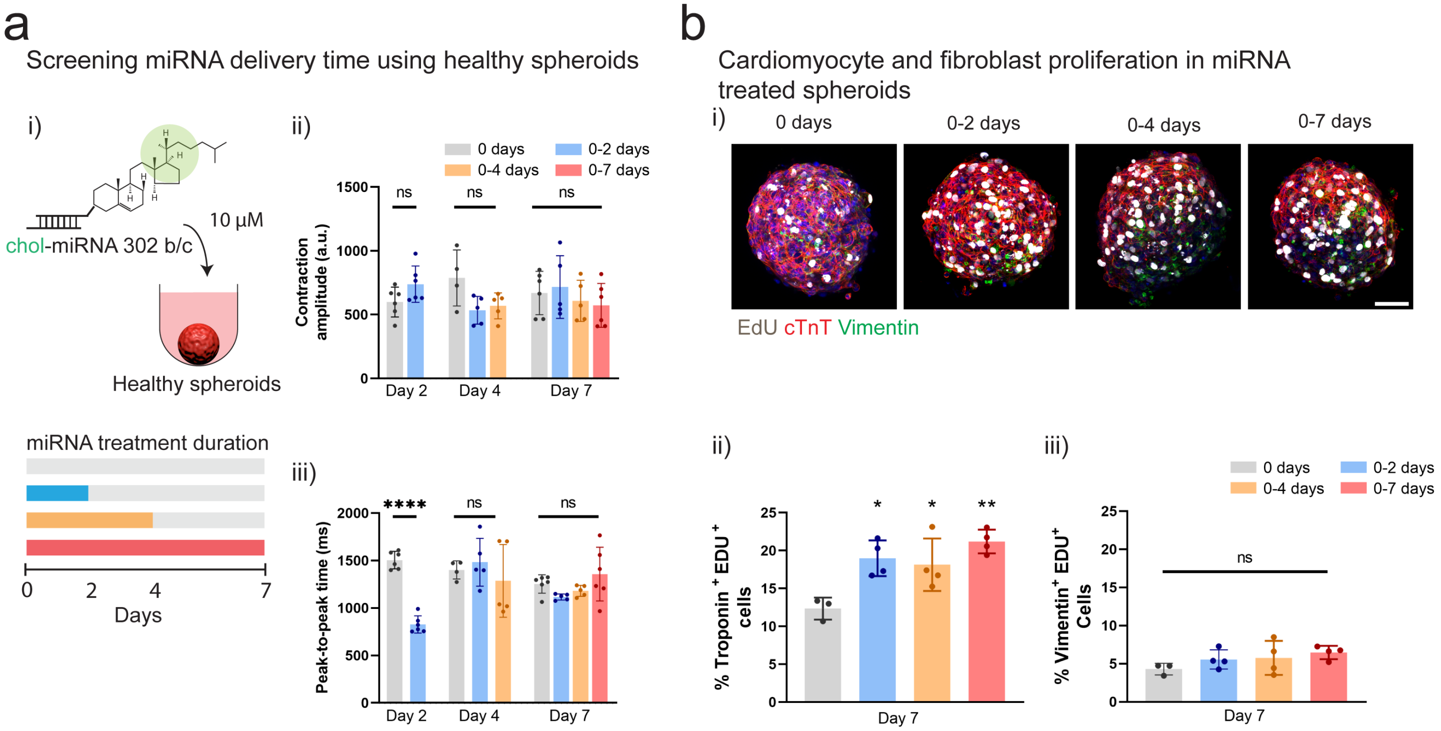

Supplementary Figure 9: miRNA screening in healthy cardiac spheroids. (a) (i) Schematic of cholesterol modified miR302 (chol-miRNA 302 b/c) delivery to healthy cardiac spheroids for 0, 0-2, 0-4, and 0-7 days. (ii) Contraction amplitude (a.u) and (iii) peak-to-peak time (ms) within healthy spheroids after 2, 4, and 7 days for each treatment period (n=6, 6, 4, 5, 5, 6, 5, 5, 6 biologically independent samples (from left to right), mean ± s.d, one-way ANOVA, (iii) day 2 - 0 vs. 0-2 days treatment $p=1.0x10^{-5}$). **(b)** (i) Immunofluorescence staining for cTnT (red; iPSC-CMs), vimentin (green; cardiac fibroblasts), and EdU (proliferation marker) in healthy spheroids at day 7 for each treatment condition. Quantification of (ii) cardiomyocyte proliferation (EdU^+ and $cTnT^+$) and (iii) fibroblast proliferation (EdU^+ and $Vimentin^+$) at day 7 (n=3, 4, 4, 4 biologically independent samples (from left to right), mean ± s.d, one-way ANOVA, (ii) 0 vs. 0-2 days treatment p=0.019, 0 vs. 0-4 days treatment p=0.040, 0 vs. 0-7 days treatment p=0.0026). Scalebar 50µm. All experiments are from a single iPSC-CM donor (donor B). (n.s. not significant, *p<0.05, **p<0.01, ****p<0.0001).

Screening miRNA delivery time using scarred microtissues

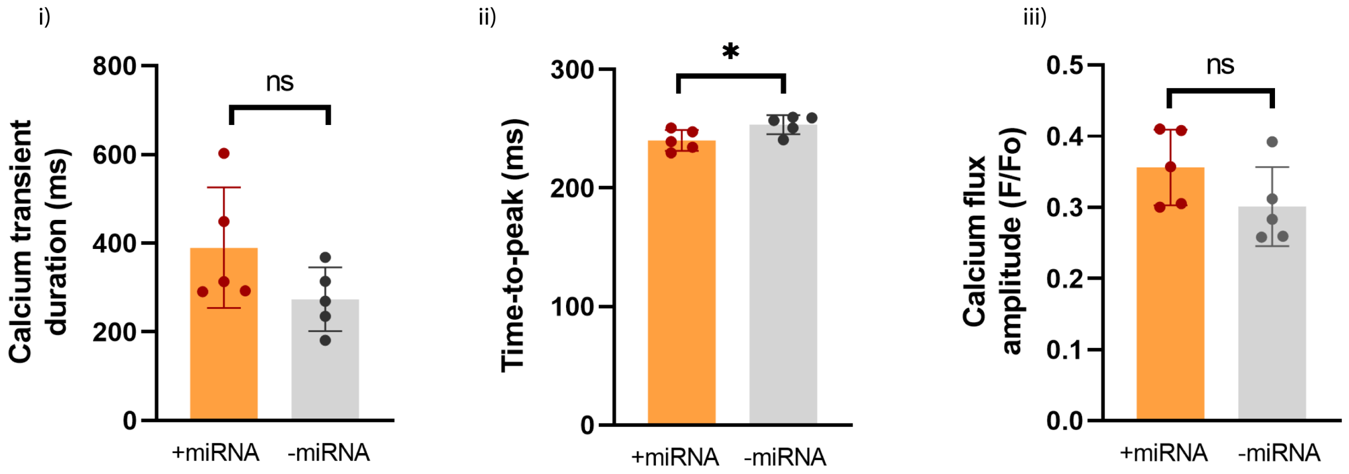

Supplementary Figure 10: Calcium activation parameters in response to miRNA treatment using scarred microtissues: Quantification of (i) calcium transient duration (ms), (ii) time-to-peak map (ms), and (iii) calcium flux amplitude (F/Fo) in scarred region of microtissues (5 days culture in support hydrogel; with and without 4 days miRNA treatment). (n=5 biologically independent samples, two-sided student t-test, (ii) p=0.037). Each data point represents the mean CTD, time-to-peak, and calcium flux amplitude in the scarred region of the microtissue. All experiments are from a single iPSC-CM donor (donor B). (*p<0.05).

Supplementary Table 1: Summary of prior studies that have used cardiomyocyte spheroids, including the source of cardiomyocytes and electrophysiology analysis used if applicable.

	Paper	Source of cardiomyocytes	Optical mapping parameters	Patch clamp parameters	Multi electrode array parameters
1	Daly et al. 2020<br>* This paper	Human iPSC-CM	Calcium activation propagation map, CT trace, CFA, CTD, Time-to-peak, activation delay		
2	(Kim et al. 2018)	Neonatal Rat Ventricular myocytes	Calcium activation propagation map, CT traces, activation delay		
3	(Zuppinger 2019)	Human iPSC-CM	CT trace, Calcium activation propagation map		
4	(Arai et al. 2018)	Human iPSC-CM	none		
5	(Ong et al. 2017)	Human iPSC-CM	AP propagation map, AP traces, CV, APD		
6	(Beauchamp et al. 2020)	Human iPSC-CM		Amplitude and resting membrane potential, AP trace, APD, Upstroke velocity (DV/DT)	
7	(Giacomelli et al. 2020)	Human iPSC-CM	CT trace, time-to-peak, decay 90%, peak-to-half decay time	AP trace, resting membrane potential, APD, APA, Velocity max, **	
8	(Mattapally et al. 2018)	Human iPSC-CM	AP propagation map, AP traces, APD, CTD		
9	(Richards et al. 2020)	Human iPSC-CM	CT traces, CT propagation map, CFA		
10	(Polonchuk et al. 2017)	Human iPSC-CM	none		
11	(Archer et al. 2018)	Human iPSC-CM	none		
12	(Lee et al. 2019)	Human ESC-CM			Averaged field potential, Field potential duration
13	(Desroches et al. 2012)	Neonatal Rat Ventricular myocytes	AP/CT trace, activation map, AP/CT duration, APD, APD map,	Recording of inward rectifier K^+ current, **	AP traces,
14	(LaBarge et al. 2019)	Human iPSC-CM	Activation propagation map, AP trace, Conduction velocity, APD		
15	(Giacomelli et al. 2017)	Human ESC-CM		AP trace, APD, APA, Diastolic membrane potential	QT and RR intervals
16	(Noguchi et al. 2016)	Neonatal Rat Ventricular myocytes	none		
17	(Tan et al. 2017)	Human iPSC-CM	CFA, CTD, CT		
18	(Richards et al. 2016)	Human iPSC-CM	none		
19	(Richards et al. 2017)	Human iPSC-CM	CFA, CTD, Time-to-peak, CT		
20	(Arai et al. 2020)	Human iPSC-CM	none		
21	(Pitaktong et al. 2019)	Human iPSC-CM	none		
22	(Cui et al. 2019)	Human iPSC-CM	CT trace, CFA, CV	AP trace, APD	
23	(Beauchamp et al. 2015)	Human iPSC-CM	CT trace, Calcium activation propagation map		
24	(Jakab et al. 2008)	Chicken embryos	none		
25	(Kelm et al. 2004)	Neonatal Rat myocytes	none		
26	(Tan et al. 2015)	Neonatal Rat myocytes + Human iPSC-CM	CT trace, CFA, time-to-peak		
27	(Figtree et al. 2017)	Neonatal Rat myocytes	none		
28	(Jiang et al. 2015)	Primate ESC and iPSC CMs	CT trace		
29	(Varzideh, Mahmoudi, and Pahlavan 2019)	Human ESC–derived cardiac progenitor cells			Field potential duration, Interspike interval (beating rate), Field potential amplitude

AP: Action potential; **APA:** Action Potential amplitude; **APD:** Action potential duration; **CFA:** Calcium flux amplitude; **CM**: Cardiomyocyte; **CT:** Calcium transient trace; **CTD:** Calcium transient duration; **CV:** Conduction velocity; **iPSC:** induced-pluripotent stem cell

** analysis was all performed on single dissociated cells taken from spheroids

Supplementary References:

Arai, Kenichi, Daiki Murata, Shoko Takao, Anna Nakamura, Manabu Itoh, Takahiro Kitsuka, and Koichi Nakayama. 2020. 'Drug response analysis for scaffold-free cardiac constructs fabricated using bio-3D printer', *Scientific Reports*, 10: 8972.

Arai, Kenichi, Daiki Murata, Ana Raquel Verissimo, Yosuke Mukae, Manabu Itoh, Anna Nakamura, Shigeki Morita, and Koichi Nakayama. 2018. 'Fabrication of scaffold-free tubular cardiac constructs using a Bio-3D printer', *PLOS ONE*, 13: e0209162.

Archer, Caroline R., Rebecca Sargeant, Jayati Basak, James Pilling, Jennifer R. Barnes, and Amy Pointon. 2018. 'Characterization and Validation of a Human 3D Cardiac Microtissue for the Assessment of Changes in Cardiac Pathology', *Scientific Reports*, 8: 10160.

Beauchamp, Philippe, Christopher B. Jackson, Lijo Cherian Ozhathil, Irina Agarkova, Cristi L. Galindo, Douglas B. Sawyer, Thomas M. Suter, and Christian Zuppinger. 2020. '3D Co-culture of hiPSC-Derived Cardiomyocytes With Cardiac Fibroblasts Improves Tissue-Like Features of Cardiac Spheroids', *Frontiers in Molecular Biosciences*, 7.

Beauchamp, Philippe, Wolfgang Moritz, Jens M. Kelm, Nina D. Ullrich, Irina Agarkova, Blake D. Anson, Thomas M. Suter, and Christian Zuppinger. 2015. 'Development and Characterization of a Scaffold-Free 3D Spheroid Model of Induced Pluripotent Stem Cell-Derived Human Cardiomyocytes', *Tissue Engineering Part C: Methods*, 21: 852-61.

Cui, Chang, Jiaxian Wang, Duoduo Qian, Jiayi Huang, Jiao Lin, Peter Kingshott, Peng-Yuan Wang, and Minglong Chen. 2019. 'Binary Colloidal Crystals Drive Spheroid Formation and Accelerate Maturation of Human-Induced Pluripotent Stem Cell-Derived Cardiomyocytes', *ACS Applied Materials & Interfaces*, 11: 3679-89.

Desroches, B. R., P. Zhang, B. R. Choi, M. E. King, A. E. Maldonado, W. Li, A. Rago, G. Liu, N. Nath, K. M. Hartmann, B. Yang, G. Koren, J. R. Morgan, and U. Mende. 2012. 'Functional scaffold-free 3-D cardiac microtissues: a novel model for the investigation of heart cells', *Am J Physiol Heart Circ Physiol*, 302: H2031-42.

Figtree, G. A., K. J. Bubb, O. Tang, E. Kizana, and C. Gentile. 2017. 'Vascularized Cardiac Spheroids as Novel 3D in vitro Models to Study Cardiac Fibrosis', *Cells Tissues Organs*, 204: 191-98.

Giacomelli, Elisa, Milena Bellin, Luca Sala, Berend J. van Meer, Leon G. J. Tertoolen, Valeria V. Orlova, and Christine L. Mummery. 2017. 'Three-dimensional cardiac microtissues composed of cardiomyocytes and endothelial cells co-differentiated from human pluripotent stem cells', *Development*, 144: 1008.

Giacomelli, Elisa, Viviana Meraviglia, Giulia Campostrini, Amy Cochrane, Xu Cao, Ruben W. J. van Helden, Ana Krotenberg Garcia, Maria Mircea, Sarantos Kostidis, Richard P. Davis, Berend J. van Meer, Carolina R. Jost, Abraham J. Koster, Hailiang Mei, David G. Míguez, Aat A. Mulder, Mario Ledesma-Terrón, Giulio Pompilio, Luca Sala, Daniela C. F. Salvatori, Roderick C. Slieker, Elena Sommariva, Antoine A. F. de Vries, Martin Giera, Stefan Semrau, Leon G. J. Tertoolen, Valeria V. Orlova, Milena Bellin, and Christine L. Mummery. 2020. 'Human-iPSC-Derived Cardiac Stromal Cells Enhance Maturation in 3D Cardiac Microtissues and Reveal Non-cardiomyocyte Contributions to Heart Disease', *Cell Stem Cell*, 26: 862-79.e11.

Jakab, K., C. Norotte, B. Damon, F. Marga, A. Neagu, C. L. Besch-Williford, A. Kachurin, K. H. Church, H. Park, V. Mironov, R. Markwald, G. Vunjak-Novakovic, and G. Forgacs. 2008. 'Tissue engineering by self-assembly of cells printed into topologically defined structures', *Tissue Engineering - Part A.*, 14: 413-21.

Jiang, B., Z. Xiang, Z. Ai, H. Wang, Y. Li, W. Ji, and T. Li. 2015. 'Generation of cardiac spheres from primate pluripotent stem cells in a small molecule-based 3D system', *Biomaterials*, 65: 103-14.

Kelm, J. M., E. Ehler, L. K. Nielsen, S. Schlatter, J. C. Perriard, and M. Fussenegger. 2004. 'Design of Artificial Myocardial Microtissues', *Tissue Engineering*, 10: 201-14.

Kim, Tae Yun, Celinda M. Kofron, Michelle E. King, Alexander R. Markes, Amenawon O. Okundaye, Zhilin Qu, Ulrike Mende, and Bum-Rak Choi. 2018. 'Directed fusion of cardiac spheroids into larger heterocellular microtissues enables investigation of cardiac action potential propagation via cardiac fibroblasts', *PLOS ONE*, 13: e0196714.

LaBarge, W., S. Mattappally, R. Kannappan, V. G. Fast, D. Pretorius, J. L. Berry, and J. Zhang. 2019. 'Maturation of three-dimensional, hiPSC-derived cardiomyocyte spheroids utilizing cyclic, uniaxial stretch and electrical stimulation', *PLOS ONE*, 14: e0219442.

Lee, Mi-Ok, Kwang Bo Jung, Seong-Jae Jo, Sung-Ae Hyun, Kyoung-Sik Moon, Joung-Wook Seo, Sang-Heon Kim, and Mi-Young Son. 2019. 'Modelling cardiac fibrosis using three-dimensional cardiac microtissues derived from human embryonic stem cells', *Journal of Biological Engineering*, 13: 15.

Mattapally, Saidulu, Wuqiang Zhu, Vladimir G. Fast, Ling Gao, Chelsea Worley, Ramaswamy Kannappan, Anton V. Borovjagin, and Jianyi Zhang. 2018. 'Spheroids of cardiomyocytes derived from human-induced pluripotent stem cells improve recovery from myocardial injury in mice', *American Journal of Physiology-Heart and Circulatory Physiology*, 315: H327-H39.

Noguchi, Ryo, Koichi Nakayama, Manabu Itoh, Keiji Kamohara, Kojirou Furukawa, Jun-ichi Oyama, Koichi Node, and Shigeki Morita. 2016. 'Development of a three-dimensional pre-vascularized scaffold-free contractile cardiac patch for treating heart disease', *The Journal of Heart and Lung Transplantation*, 35: 137-45.

Ong, Chin Siang, Takuma Fukunishi, Huaitao Zhang, Chen Yu Huang, Andrew Nashed, Adriana Blazeski, Deborah DiSilvestre, Luca Vricella, John Conte, Leslie Tung, Gordon F. Tomaselli, and Narutoshi Hibino. 2017. 'Biomaterial-Free Three-Dimensional Bioprinting of Cardiac Tissue using Human Induced Pluripotent Stem Cell Derived Cardiomyocytes', *Scientific Reports*, 7: 4566.

Pitaktong, Isaree, Cecillia Lui, Justin Lowenthal, Gunnar Mattson, Wei-Hung Jung, Yang Bai, Enoch Yeung, Chin Siang Ong, Yun Chen, Sharon Gerecht, and Narutoshi Hibino. 2019. 'Early Vascular Cells Improve Microvascularization Within 3D Cardiac Spheroids', *Tissue Engineering Part C: Methods*, 26: 80-90.

Polonchuk, Liudmila, Mamta Chabria, Laura Badi, Jean-Christophe Hoflack, Gemma Figtree, Michael J. Davies, and Carmine Gentile. 2017. 'Cardiac spheroids as promising in vitro models to study the human heart microenvironment', *Scientific Reports*, 7: 7005.

Richards, Dylan J., Robert C. Coyle, Yu Tan, Jia Jia, Kerri Wong, Katelynn Toomer, Donald R. Menick, and Ying Mei. 2017. 'Inspiration from heart development: Biomimetic development of functional human cardiac organoids', *Biomaterials*, 142: 112-23.

Richards, Dylan J., Yang Li, Charles M. Kerr, Jenny Yao, Gyda C. Beeson, Robert C. Coyle, Xun Chen, Jia Jia, Brooke Damon, Robert Wilson, E. Starr Hazard, Gary Hardiman, Donald R. Menick, Craig C. Beeson, Hai Yao, Tong Ye, and Ying Mei. 2020. 'Human cardiac organoids for the modelling of myocardial infarction and drug cardiotoxicity', *Nature Biomedical Engineering*, 4: 446-62.

Richards, Dylan J., Yu Tan, Robert Coyle, Yang Li, Ruoyu Xu, Nelson Yeung, Arran Parker, Donald R. Menick, Bozhi Tian, and Ying Mei. 2016. 'Nanowires and Electrical Stimulation Synergistically Improve Functions of hiPSC Cardiac Spheroids', *Nano Letters*, 16: 4670-78.

Tan, Y., D. Richards, R. Xu, S. Stewart-Clark, S. K. Mani, T. K. Borg, D. R. Menick, B. Tian, and Y. Mei. 2015. 'Silicon nanowire-induced maturation of cardiomyocytes derived from human induced pluripotent stem cells', *Nano Letters*, 15: 2765-72.

Tan, Yu, Dylan Richards, Robert C. Coyle, Jenny Yao, Ruoyu Xu, Wenyu Gou, Hongjun Wang, Donald R. Menick, Bozhi Tian, and Ying Mei. 2017. 'Cell number per spheroid and electrical conductivity of nanowires influence the function of silicon nanowired human cardiac spheroids', *Acta Biomater*, 51: 495-504.

Varzideh, F., E. Mahmoudi, and S. Pahlavan. 2019. 'Coculture with noncardiac cells promoted maturation of human stem cell–derived cardiomyocyte microtissues', *Journal of Cellular Biochemistry*, 120: 16681-91.

Zuppinger, C. 2019. "Measurement of contractility and calcium release in cardiac spheroids." In *Methods in Molecular Biology*, 41-52.
