# Supplementary material for: 3D bioprinting of high cell-density heterogeneous tissue models through spheroid fusion within self-healing hydrogels
Source: Nat Commun. 2021 Feb 2;12:753. doi: 10.1038/s41467-021-21029-2 (PMC7854667; doi:10.1038/s41467-021-21029-2)
Supplement: Supplementary file 2 — Description of Additional Supplementary Files [file 41467_2021_21029_MOESM2_ESM.pdf]

**Description of Additional Supplementary Files**

**Supplementary Movie 1: 3D bioprinting of spheroids.** Movie and cartoon of the 1) aspiration of a spheroid, 2) transfer of the spheroid into a support gel, and 3) translation and deposition of the spheroid into the support gel into a defined pattern.

**Supplementary Movie 2: Shear-thinning and self-healing support gel during spheroid translation.** Fluorescent microscopy of the support gel containing fluorescent beads during the translation of a spheroid through the gel, illustrating gel displacement and healing.

**Supplementary Movie 3: Calcium imaging of spheroids.** Calcium imaging of "healthy" and "scarred" spheroids containing varied ratios of iPSC-derived cardiomyocytes and cardiac fibroblasts.

**Supplementary Movie 4: Cardiac microtissue contractility.** Light microscopy of cardiac microtissues formed from 8 spheroids after spheroid fusion – containing either 8 "healthy" spheroids or 7 "healthy" spheroids and 1 "scarred" spheroid to mimic healthy myocardium and myocardium after infarction, respectively.

**Supplementary Movie 5: Calcium imaging of cardiac microtissues.** Calcium imaging of microtissues containing either 8 "healthy" spheroids or 7 "healthy" spheroids and 1 "scarred" spheroid to mimic healthy myocardium and myocardium after infarction, respectively. "Scarred" spheroid shown in the center of images.
