# Supplementary material for: 3D bioprinting of high cell-density heterogeneous tissue models through spheroid fusion within self-healing hydrogels
Source: Nat Commun. 2021 Feb 2;12:753. doi: 10.1038/s41467-021-21029-2 (PMC7854667; doi:10.1038/s41467-021-21029-2)
Supplement: Supplementary file 8 — Reporting Summary [file 41467_2021_21029_MOESM8_ESM.pdf]

nature research

Corresponding author(s): J.A. Burdick

Last updated by author(s): Dec 9, 2020

Reporting Summary

Nature Research wishes to improve the reproducibility of the work that we publish. This form provides structure for consistency and transparency in reporting. For further information on Nature Research policies, see our Editorial Policies and the Editorial Policy Checklist.

Statistics

For all statistical analyses, confirm that the following items are present in the figure legend, table legend, main text, or Methods section.

n/a	Confirmed	
☐	☒	The exact sample size (*n*) for each experimental group/condition, given as a discrete number and unit of measurement
☐	☒	A statement on whether measurements were taken from distinct samples or whether the same sample was measured repeatedly
☐	☒	The statistical test(s) used AND whether they are one- or two-sided<br>*Only common tests should be described solely by name; describe more complex techniques in the Methods section.*
☒	☐	A description of all covariates tested
☒	☐	A description of any assumptions or corrections, such as tests of normality and adjustment for multiple comparisons
☐	☒	A full description of the statistical parameters including central tendency (e.g. means) or other basic estimates (e.g. regression coefficient) AND variation (e.g. standard deviation) or associated estimates of uncertainty (e.g. confidence intervals)
☐	☒	For null hypothesis testing, the test statistic (e.g. *F*, *t*, *r*) with confidence intervals, effect sizes, degrees of freedom and *P* value noted<br>*Give P values as exact values whenever suitable.*
☒	☐	For Bayesian analysis, information on the choice of priors and Markov chain Monte Carlo settings
☒	☐	For hierarchical and complex designs, identification of the appropriate level for tests and full reporting of outcomes
☒	☐	Estimates of effect sizes (e.g. Cohen's *d*, Pearson's *r*), indicating how they were calculated

Our web collection on statistics for biologists contains articles on many of the points above.

Software and code

Policy information about availability of computer code

Data collection	Bruker DMX 360 (Acquisition of NMR spectra)<br>Leica (LAS X) (Confocal fluorescence imaging)<br>Metamorph (V7.8.4.0) (Interface used for Nikon spinning disk confocal calcium imaging)<br>Rheology advantage (V5.8.2), TA instruments (Rheological data collection)
Data analysis	Graphpad prism (V8.4.1) (Plotting, statistical analysis)<br>Solidworks CAD software (v26)<br>Image J (V 1.53)(Confocal image analysis; cell proliferation, fusion area, printing precision measurements)<br>Microsoft excel (V16) (Data handling)<br>Adobe illustrator (V2020) (Figure preparation)<br>Topsin (V4.0.9) (NMR analysis)<br>Musclemotion (Contraction analysis) (https://github.com/l-sala/MUSCLEMOTION)<br>Electromap (Calcium imaging analysis) (https://github.com/CXO531/ElectroMap)

For manuscripts utilizing custom algorithms or software that are central to the research but not yet described in published literature, software must be made available to editors and reviewers. We strongly encourage code deposition in a community repository (e.g. GitHub). See the Nature Research guidelines for submitting code & software for further information.

Data

Policy information about availability of data

All manuscripts must include a data availability statement. This statement should provide the following information, where applicable:

- Accession codes, unique identifiers, or web links for publicly available datasets
- A list of figures that have associated raw data
- A description of any restrictions on data availability

All data supporting the results in this study are available within the article and its supplementary information. The broad range of raw datasets acquired and analyzed are available from the corresponding author on reasonable request. Source data are provided with this paper.

Field-specific reporting

Please select the one below that is the best fit for your research. If you are not sure, read the appropriate sections before making your selection.

☒ Life sciences ☐ Behavioural & social sciences ☐ Ecological, evolutionary & environmental sciences

For a reference copy of the document with all sections, see nature.com/documents/nr-reporting-summary-flat.pdf

Life sciences study design

All studies must disclose on these points even when the disclosure is negative.

Sample size	Power analysis of sample size was not done prior to experimentation. The study detailed the development of a new bioprinting method, and as there was no prior data, a statistical determination of sample size was not possible. For calcium and contraction imaging experiments, n was based on previous papers using iPSC-CMs (Wang et al., 10.1021/acscentsci.9b00052; Richards et al., 10.1038/s41551-020-0539-4). Given the reproducibility within and across experiments we concluded the sample sizes in our experiment were sufficient.
Data exclusions	3 data points were excluded from the analysis in figure 4c. The data points are noted as x's on the graph. The data points were excluded because no significant calcium activation event was observed for these spheroids, which is indicated in the figure caption.
Replication	The spheroid printing method using self-healing hydrogels (Figure 1, 2), and subsequent fusion into microtissues (Figure 3), was performed using 1 MSC donor as described in the figure legends. Cardiac spheroid and microtissue experiments were performed using iPSC-CMs from two donors (Figure 4 & 5, donor A; Figure 6, Donor B) as described in the figure legends. Validation of spheroid fusion into microtissues across different cell types & donors demonstrates the reproducibility of our bioprinting method.
Randomization	Randomization methods did not apply to this study as there were no clinical populations or patients involved. All quantification of fluorescence images involved analyzing whole samples, and no regional randomizations were applicable (i.e. cell number quantifications were performed across the entire spheroid, calcium activation analysis encompasses the whole microtissue).
Blinding	The investigators were not blinded to allocation during experiments and outcome measurements. Our data sets are based on objectively measurable data (fluorescent intensity, fluorescence area). Blinding does not affect these data values.

Reporting for specific materials, systems and methods

We require information from authors about some types of materials, experimental systems and methods used in many studies. Here, indicate whether each material, system or method listed is relevant to your study. If you are not sure if a list item applies to your research, read the appropriate section before selecting a response.

Materials & experimental systems

n/a	Involved in the study	
☐	☒	Antibodies
☐	☒	Eukaryotic cell lines
☒	☐	Palaeontology and archaeology
☒	☐	Animals and other organisms
☒	☐	Human research participants
☒	☐	Clinical data
☒	☐	Dual use research of concern

Methods

n/a	Involved in the study	
☒	☐	ChIP-seq
☒	☐	Flow cytometry
☒	☐	MRI-based neuroimaging

Antibodies

Antibodies used	Primary antibodies:<br>Cardiac troponin-t (Thermofisher MA5-12960), clone 13-11, Lot Number: UG2798731<br>Vimentin (Thermofisher MA5-16409), clone SP20, Lot Number: UJ2870531<br>Anti-sarcomeric alpha actinin antibody (abcam 137346), Lot Number: GR3307040-5<br>Connexin 43 (Thermofisher 71-0700), Lot Number: UG289624<br>Secondary antibodies:<br>Alexa Fluor 488 (abcam 150077), Lot 1858182<br>Alexa Fluor 594 (abcam 150116), Lot 1922849<br>Alexa Fluor 647 (abcam 150079), Lot 1696456
Validation	- Cardiac troponin-t (Thermofisher MA5-12960) https://assets.thermofisher.com/TFS-Assets/LSG/certificate/Certificates-of-Analysis/MA512960_UG2798731.PDF, (Burridge et al, 10.1038/nm.4087) (Titmarsh et al., 10.1038/srep24637)<br>- Vimentin (Thermofisher MA5-16409), https://assets.thermofisher.com/TFS-Assets/LSG/certificate/Certificates-of-Analysis/MA516409_UJ2870531.PDF, (Buono et al. 10.3390/cells9071733)<br>- Anti-sarcomeric alpha actinin antibody (abcam 137346), https://www.abcam.com/sarcomeric-alpha-actinin-antibody-ab137346.html , (Kim et al., 10.1038/s41467-019-14019-y)<br>- Connexin 43 (Thermofisher 71-0700), https://assets.thermofisher.com/TFS-Assets/LSG/certificate/Certificates-of-Analysis/710700_UG289624.PDF, (Crestani et al. 10.1016/j.bbrc.2020.09.021, Lucero et al. 10.1038/s41598-020-63336-6)

Eukaryotic cell lines

Policy information about cell lines

Cell line source(s)	No eukaryotic cell lines were used in this study.<br>The primary cells and commercially derived iPSC-cardiomyocytes used are:<br>Human induced pluripotent stem cell-derived cardiomyocytes (hiPSC-CMs),<br>iCell Cardiomyocytes iPSC donor 01434, Cellular Dynamics International-CDI, Madison, WI, USA<br>iCell Cardiomyocytes iPSC donor 11713, Cellular Dynamics International-CDI, Madison, WI, USA<br><br>Human cardiac fibroblasts were purchased from Promocell (c-12375)<br>Human MSCs were isolated from fresh bone marrow purchased from Lonza (1M-125)
Authentication	Cells were authenticated by the vendor, and we did not further authenticate during experimentation.
Mycoplasma contamination	Cells tested negative for mycoplasma contamination according to the manufacturer, and were not tested for mycoplasma contamination during our experiments.
Commonly misidentified lines<br>(See ICLAC register)	No commonly misidentified cell lines were used.
